# Development of the HTRF assay to evaluate the auxin‐induced binding between TIR1 and IAA7

**DOI:** 10.1111/nph.71084

**Published:** 2026-03-11

**Authors:** Jekson Robertlee, Shinya Hagihara

**Affiliations:** ^1^ RIKEN Center for Sustainable Resource Science 2‐1 Hirosawa Wako Saitama 351‐0198 Japan

**Keywords:** auxin, auxin analogs, auxin co‐receptors, HTRF, IAA7, plant thermosensor, protein–protein interaction, TIR1

## Abstract

- Auxin plays diverse roles in plant growth and development, including sensing environmental changes. Quantifying the interaction between auxin coreceptors provides the molecular basis for cells to sense and adapt to environmental cues. Although several assays are available, a more high‐throughput method is necessary to efficiently evaluate the auxin‐induced binding of coreceptors.
- We developed a homogeneous time‐resolved fluorescence (HTRF) assay to quantitatively measure the binding between the *Arabidopsis thaliana* TRANSPORT INHIBITOR RESPONSE 1 (TIR1) and indole‐3‐acetic acid 7 (IAA7) auxin coreceptor proteins.
- The HTRF assay provides a rapid analysis with sensitivity similar to the enzyme‐linked immunosorbent assay. We demonstrated its effectiveness by analyzing the potency of several auxin analogs to induce binding between TIR1 and IAA7. We also found that a mild increase in temperature impairs the binding activity of TIR1 to IAA7.
- This method provides a rapid and robust tool to evaluate the auxin‐induced binding between TIR1 and Aux/IAA auxin coreceptors. A similar strategy may also be applicable to study other plant hormone heterodimer coreceptors.

## Introduction

Auxin, one of the most studied plant hormones, has a general role in plant growth and development (Teale *et al*., 2006). Auxin is also involved in adapting the plant body to various environmental changes by sensing drought and salinity stresses, light, heat, and gravity (Jing *et al*., 2023). Auxin triggers signaling through its interaction with coreceptor proteins TRANSPORT INHIBITOR RESPONSE 1 (TIR1)/AUXIN‐SIGNALING F‐BOX (AFB) and AUXIN/INDOLE‐3‐ACETIC ACID (Aux/IAA). The resulting complex formation promotes downstream processes, such as the degradation of Aux/IAA proteins and the regulation of auxin‐related genes (Gray *et al*., 2001; Dharmasiri *et al*., 2005; Kepinski & Leyser, 2005; Tan *et al*., 2007). A variety of naturally occurring auxins, such as IAA, 4‐chloroindole‐IAA (4‐Cl‐IAA), and phenylacetic acid (PAA), induce an auxin response (Korasick *et al*., 2013; Simon *et al*., 2013). In addition to these compounds, many synthetic plant growth regulators with auxin‐like activities are categorized as auxin analogs.

Direct quantification of the interaction between auxin analogs and coreceptors provides essential information for understanding how auxin regulates plant growth and development. The first technique developed to assess auxin activity was a transcriptional reporter assay, which uses an auxin‐responsive promoter to express β‐glucuronidase (Ulmasov *et al*., 1997; Sabatini *et al*., 1999). Post‐transcriptional reporter assays were then developed to monitor the real‐time distribution of auxin in the plant. In these systems, a fluorescent protein is fused in‐frame to the Aux/IAA auxin‐interaction domain (termed domain II; DII), and the auxin activity is reflected by a reduction in fluorescence (Brunoud *et al*., 2012; Liao *et al*., 2015). More recently, a fluorescence resonance energy transfer‐based biosensor has also been developed to visualize fluctuations in IAA levels (Herud‐Sikimić *et al*., 2021). These *in planta* systems have been widely used and are indispensable for understanding the distribution and function of auxins (Jedličková *et al*., 2022). The yeast two‐hybrid (Y2H) assay provides an alternative method to evaluate the auxin‐inducible interaction between coreceptors outside of plant cells (Prigge *et al*., 2010) and has been used for simultaneous analyses of multiple auxin analogs (Uchida *et al*., 2018; Yamada *et al*., 2018). Although Y2H can provide some insights into the potency of each ligand to induce the binding between auxin coreceptors, the output is limited to a binary assessment at the tested concentrations. Moreover, these *in planta* and *in cell* assays indirectly reflect the binding potency of an auxin analog to its coreceptors because many *in vivo* factors, such as ligand transport, metabolism, and downstream signaling, may affect the analysis.

In terms of the *in vitro* evaluation system of auxin coreceptors binding, several systems have been reported, including the pull‐down assay (Gray *et al*., 2001; Dharmasiri *et al*., 2003), radioligand binding assay, surface plasmon resonance (SPR) assay (Calderón Villalobos *et al*., 2012), and AlphaScreen assay (Cao *et al*., 2022). Among these, the radioligand assay offers the highest sensitivity, but the requirement of radioisotope‐labeled ligands often limits its use. Alternatively, the *in vitro* pull‐down assay can be performed without specialized equipment (Uchida *et al*., 2018). The SPR assay provides a highly sensitive, real‐time binding approach to evaluate nonlabeled auxin analogs and has been used in recent years (Lee *et al*., 2014; Uzunova *et al*., 2016; Prusinska *et al*., 2022; Sun *et al*., 2024). The AlphaScreen assay is a mix‐and‐read format suitable for high‐throughput analysis with high sensitivity. However, photobleaching of the donor beads and its single‐read limitation make it less ideal for monitoring dynamic binding changes over time (Eglen *et al*., 2008).

The *Arabidopsis* genome encodes 23 AUX/IAA and 6 TIR1/AFB proteins, and the combination of these coreceptors contributes to the spatiotemporal regulation of auxin signaling. Quantitative analysis of how natural and synthetic auxins interact with each coreceptor combination will facilitate the understanding and manipulation of auxin‐related phenomena with high precision. To achieve this, a high‐throughput analytical method for evaluating auxin coreceptor interactions is necessary. In this study, we applied enzyme‐linked immunosorbent assay (ELISA) and homogeneous time‐resolved fluorescence (HTRF) assays to evaluate the binding between TIR1 and IAA7 *in vitro*. Our results show similar sensitivities for both ELISA and HTRF assays, and they are comparable to other established techniques. The HTRF assay provides a more rapid analysis than the ELISA and allows for multiple measurements over time. Using the HTRF system, we analyzed the potency of several known auxin analogs to induce the binding between TIR1 and IAA7. We also found that TIR1 loses its binding activity to IAA7 after a brief exposure to a mild temperature increase. The strategy presented in this study will provide a convenient and straightforward method for evaluating auxin analogs.

## Materials and Methods

### Plasmid construction

The *Arabidopsis thaliana* (L.) Heynh. TIR1 and IAA7 genes were used in this study. Plant vector cloning was performed using the GoldenBraid system, a gift from Diego Orzaez (Addgene kit no. 1000000076). The procedures for domesticating the target genes for GoldenGate‐compatible cloning followed the methods described previously by the Orzaez group (Sarrion‐Perdigones *et al*., 2013; Vazquez‐Vilar *et al*., 2017). For bacterial expression, the pGEX‐2T vector (Cytiva, Marlborough, MA, USA) was used. Recombinant plasmid maps are provided in Supporting Information Notes S1, and the integrity of all cloned regions was confirmed by sequencing analysis.

### TIR1 expression in *N. benthamiana*

Wild‐type (WT) *Nicotiana benthamiana* (Domin) plants, grown in a temperature‐controlled glasshouse on the RIKEN Wako campus, were used for transient expression. For agroinfiltration, electro‐competent *Agrobacterium tumefaciens* LBA4404 (Takara, Shiga, Japan) was used. The transformed *Agrobacterium* was cultured in LB medium supplemented with 100 μg ml^−1^ rifampicin, streptomycin, and kanamycin. The agroinfiltration procedure followed the methods as previously described (Norkunas *et al*., 2018). Briefly, cultured *Agrobacterium* cells were washed four times with agroinfiltration buffer (10 mM MgCl2, 10 mM MES buffer pH 5.5, and 0.3 mM acetosyringone) and incubated at room temperature in the dark for *c*. 1 h. The cultures were then adjusted to an OD_600_ of 0.5 before infiltrating the leaves of one‐ to two‐month‐old *N. benthamiana* plants. All samples were cotransformed with the P19 protein to improve expression yield. The infiltrated leaves were harvested after 10 d, flash‐frozen in liquid nitrogen, and stored at −80°C.

### TIR1 purification from *N. benthamiana* leaves

Leaves (50 g) were ground into a fine powder in liquid nitrogen. All protein extraction and purification procedures were performed at 4°C or on ice. The fine powder was thoroughly mixed with 150 ml of freshly prepared extraction buffer, which consisted of 1× tris‐buffered saline (TBS, Nippon Gene, Tokyo, Japan, 317‐90371), 150 mM NaCl (Nacalai Tesque, Kyoto, Japan, 31 334‐51), 0.5% Triton X‐100 (Sigma, CAS: 9036‐19‐5), 0.05% 2‐Mercaptoethanol (Nacalai Tesque, 21438–82), and 1× protease inhibitor cocktail (Nacalai Tesque, 03969‐21). The mixture was centrifuged at 18 000 ***g*** for an hour to remove the cell debris, and the supernatant was collected for affinity tag purification. The His‐tagged protein in the supernatant was purified using a gravity‐flow column (Bio‐Rad, Hercules, CA, USA, 7321010) containing TALON metal affinity resin (Takara). The resin was washed with 100 times the bead volume. The washing buffer consisted of 50 mM phosphate buffer solution (pH 7.4; Nacalai Tesque 37244‐35), 450 mM NaCl, 5 mM imidazole (Nacalai Tesque, 19004‐35), and 0.5% Triton X‐100. The resin was then washed three times with 10 ml of washing buffer without detergent and eluted with 1× TBS containing 180 mM imidazole.

### IAA7 expression in *Escherichia coli* and its purification

The *Escherichia coli* cells harboring the pGEX‐2T plasmid for IAA7 expression were cultivated overnight in LB medium containing 100 μg ml^−1^ ampicillin at 37°C. For protein expression, the culture was scaled up the following day. Once the OD_600_ reached *c*. 0.8, the culture was cooled on ice. Isopropyl‐β‐d‐1‐thiogalactopyranoside (IPTG, Fujifilm Wako Pure Chemical, Osaka, Japan, 092‐05324) was then added to a final concentration of 0.4 mM, and the culture was incubated overnight at 15°C. Cells were harvested by centrifugation at 4000 ***g*** and stored at −80°C. The cell pellet was resuspended in 20 ml of freshly prepared lysis buffer and sonicated. The lysis buffer consisted of 1× phosphate‐buffered saline (PBS, Nacalai Tesque, 11482‐15), 500 mM NaCl, 1 mM dithiothreitol (DTT, Nacalai Tesque, 14128‐04), 0.005 mg mL^−1^ DNAse (Fujifilm Wako Pure Chemical, 047‐26771), 1 mg mL^−1^ Lysozyme (Nacalai Tesque, 19499–91), and 1× protease inhibitor cocktail (Nacalai Tesque, 04080–11). The lysate was centrifuged at 18 000 ***g*** for 1 h to remove cell debris, and the supernatant was used for affinity tag purification. The GST‐tagged protein was purified from the supernatant using COSMOGEL(R) GST‐Accept (Nacalai Tesque, 09277–72) in a gravity‐flow column (Bio‐Rad, 7321 010), and the resin was washed with 100 times the bead volume. The washing buffer consisted of 1× PBS, 1 mM DTT, and 0.05% Triton X‐100 (Sigma, CAS 9036‐19‐5). The resin was washed three times with 10 ml of washing buffer without detergent and eluted using 1× TBS containing 10 mM glutathione (reduced form, Nacalai Tesque, 08786–74).

### Buffer exchange, protein assay, and storage

The eluted protein was first filtered through a 0.22‐μm membrane filter (MilliporeSigma, Burlington, MA, USA, SLGVR33RS). For buffer exchange, the filtered protein was transferred to a centrifugal filter device and exchanged with 1× TBS for four rounds. A 30‐kDa MWCO centrifugal filter (MiliporeSigma, Amicon‐15 UFC903024) was used for TIR1, while a 10‐kDa MWCO filter (MiliporeSigma, Amicon‐15 UFC901024) was used for IAA7. Protein concentrations were determined using a Coomassie brilliant blue assay (Nacalai Tesque, 29449‐44), which was standardized with bovine serum albumin (Nacalai Tesque, 00653‐31). The proteins were then aliquoted into small volumes, flash‐frozen in liquid nitrogen, and stored at −80°C. The protein analysis was performed using an SDS‐PAGE strain‐free gel (Bio‐Rad, no. 4568096) and visualized using the ChemiDoc MP system (Bio‐Rad).

### ELISA assay

The ELISA procedure was performed on ice, with all subsequent incubations at 4°C unless otherwise noted. A saturating amount of GST_IAA7 protein (100 μl, 2200 nM in TBS buffer) was incubated on the 96‐well plate (Immuno MaxiSorp, #442404) for 3 h to immobilize the protein. The plate was then washed three times with TBS‐T (TBS buffer supplemented with 0.05% Tween 20) and blocked with 300 μl of 5% milk in TBS buffer for 1 h at room temperature. Following another wash with TBS‐T, 200 μl of FLAG_TIR1 or FLAG_TIR1^F79A^ (150 nM) was added to each well. Various ligand concentrations (1% DMSO) were then added, and the plate was incubated for 1.5 h. After washing three times, 100 μl of the primary antibody (FLAG‐antibody, MBL, M185‐3 l, 10 000× dilution in 1× TBS buffer with 1% milk) was added and incubated for 1 h. A subsequent wash was performed, and 100 μl of the secondary horseradish peroxidase (HRP) conjugated antibody (Cytiva, NA931VS, 10 000× dilution in TBS buffer with 1% milk) was added and incubated for 1 h. During both primary and secondary antibody incubations, the corresponding auxin concentration (1% dimethyl sulfoxide, DMSO) was maintained in each well to retain bound TIR1. After another wash, the HRP substrate (ATTO, Tokyo, Japan, WSE‐7145) was added according to the manufacturer's instructions. The signal was then measured using a plate reader (SPARK; Tecan, Männedorf, Switzerland).

### HTRF assay

The HTRF reagents anti‐FLAG Tb‐Conjugate (Revvity, Waltham, MA, USA, 61FGBTLA, lot 01A) and anti‐GST d2‐conjugate (Revvity, 61GSTDLA, lot 16RA) were used according to the manufacturer's instructions. The HTRF buffer consisted of 1× TBS, 1 mM DTT, and 0.05% Triton X‐100. Protein concentrations were as indicated in the figures or the Results section. The assay was prepared as a master mix; 20 μl was dispensed into each well of a 384‐well plate (PerkinElmer, Waltham, MA, USA, 6057 340) before adding the ligand (1% DMSO). The plate was shaken for 10 min at 700 rpm at the indicated temperature using an Eppendorf ThermoMixer C equipped with a ThermoTop. The plate was then analyzed with a Tecan SPARK plate reader that is compatible with the HTRF assay. The measurement setup was no lid, no humidity cassette, and in a smooth mode. The parameters were as follows: mirror dichroic 510, 75 flashes, 200 μs integration time, 100 μs lag time, and a settle time of 0 ms. The Z‐position was optimized using a well containing 100 μM IAA, which was expected to produce one of the brightest acceptor signals. Two sequential measurements were performed. The donor excitation was at 340 (30) nm with emission at 620 (10) nm, and the gain was set at 228. The acceptor excitation was at 340 (30) nm with emission at 665 (10) nm, and the gain was set at 230.

### Data analysis

The HTRF ratio was calculated by dividing the time‐resolved fluorescence signal from the acceptor (665 nm) by that of the donor (620 nM). The half‐maximal effective concentration (EC_50_) of the ligand required to induce binding between TIR1 and IAA7 was determined by nonlinear regression of the ELISA or HTRF signal. Nonlinear regression was performed using the ‘[Agonist] vs response ‐‐ Variable slope (four parameters)’ equation built into the GraphPad Prism 10 software. The equation for the four‐parameter fit is as follows:

$$Y=\text{Bottom}+\frac{\left(\mathrm{Top}-\text{Bottom}\right)\times {Χ}^{\text{Hill}}}{{\mathrm{EC}}_{50}^{\text{Hill}}+{Χ}^{\text{Hill}}}$$

where *X* is the ligand concentration, *Y* is the response (HTRF ratio). Top is the maximum response. Bottom is the minimum response (baseline). EC_50_ is the half‐maximal effective concentration (in the same units as *X*). Hill is the Hill coefficient. For auxin analogs that did not reach the maximum value of the control ligand (IAA or 5‐ada‐IAA), the predicted EC_50_ was calculated by constraining the Top value to be equal to the maximum value of the control ligand.

## Results

### Protein expression and ELISA binding assay of TIR1 to IAA7

To develop a straightforward assay system for evaluating the auxin‐induced binding between TIR1 and IAA7, a sufficient amount of active protein is needed. The GST‐tagged IAA7 protein was expressed in *E. coli* and purified using glutathione agarose as previously reported (Dharmasiri *et al*., 2005). On the other hand, TIR1 has been expressed in insect suspension cells (Tan *et al*., 2007; Calderón Villalobos *et al*., 2012; Lee *et al*., 2014; Cao *et al*., 2022; Prusinska *et al*., 2022) and a cell‐free translation system (Uchida *et al*., 2018). In this study, we used a transient expression system in *N. benthamiana* to produce the TIR1 protein. Throughout this study, we expressed the TIR1 with E12K and E15K substitutions because these mutations have been known to stabilize TIR1 in the Arabidopsis plants (Yu *et al*., 2015). We obtained *c*. 160–280 μg of the affinity‐tagged purified protein from 50 g of fresh‐weight leaves, and the protein size was confirmed by SDS‐PAGE analysis (Fig. S1).

**Fig. 1:**
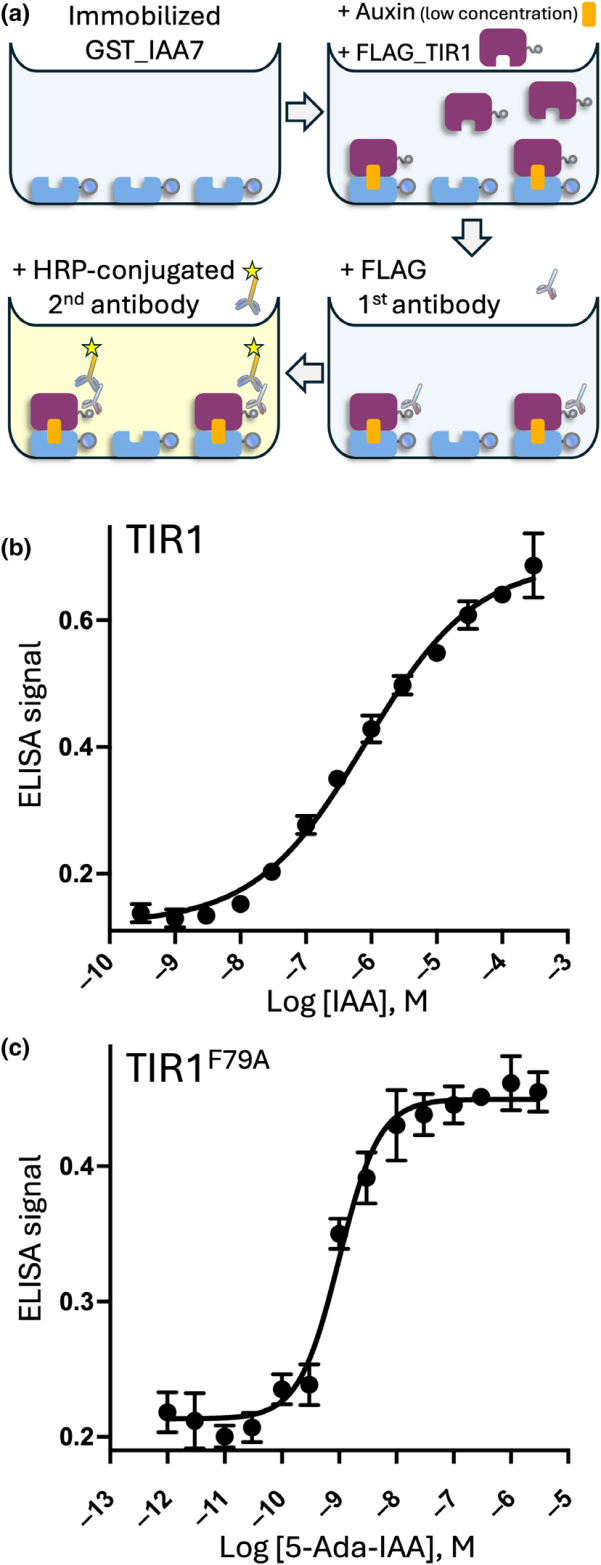
Enzyme‐linked immunosorbent assay (ELISA) to evaluate the binding between TRANSPORT INHIBITOR RESPONSE 1 (TIR1) and indole‐3‐acetic acid 7 (IAA7). (a) Schematic illustration of the ELISA. (b) The concentration–response curve shows the binding assay between TIR1 and IAA7 coreceptors in the presence of IAA. The half‐maximal effective concentration (EC_50_) was determined to be 0.81 μM, 95% confidence interval (95% CI) = 0.5–1.5 μM. (c) The concentration–response curve shows the binding between TIR1^F79A^ and IAA7 co‐receptors in the presence of 5‐ada‐IAA. EC_50_ was determined to be 0.99 nM, 95% CI = 0.78–1.3 nM. Data are presented as the mean ± SD of triplicates.

We first employed the ELISA binding assay (Fig. 1a) to confirm whether auxin can induce the binding of the purified TIR1 to the IAA7 coreceptor. We observed that the ELISA signal increased with increasing IAA concentration (Fig. 1b). Nonlinear regression showed a calculated EC_50_ value of 0.81 μM. It should be noted that the binding activity of TIR1 produced using a transient *N. benthamiana* expression system had not been previously reported. We also evaluated the IAA7 binding of the TIR1 variant, the F79A‐substituted TIR1, for which binding to Aux/IAA is strongly induced by the synthetic ligand 5‐ada‐IAA (Yamada *et al*., 2018). The EC_50_ of 5‐ada‐IAA to induce the binding of TIR1^F79A^ to IAA7 was 0.99 nM (Fig. 1c), which is considerably lower than the EC_50_ for natural IAA‐induced TIR1‐IAA7 complex formation. 5‐ada‐IAA showed higher potency in the Y2H assay than that in our ELISA, likely due to its high cellular permeability (Yamada *et al*., 2018).

### HTRF assay to evaluate the binding between TIR1 and IAA7

**Fig. 2:**
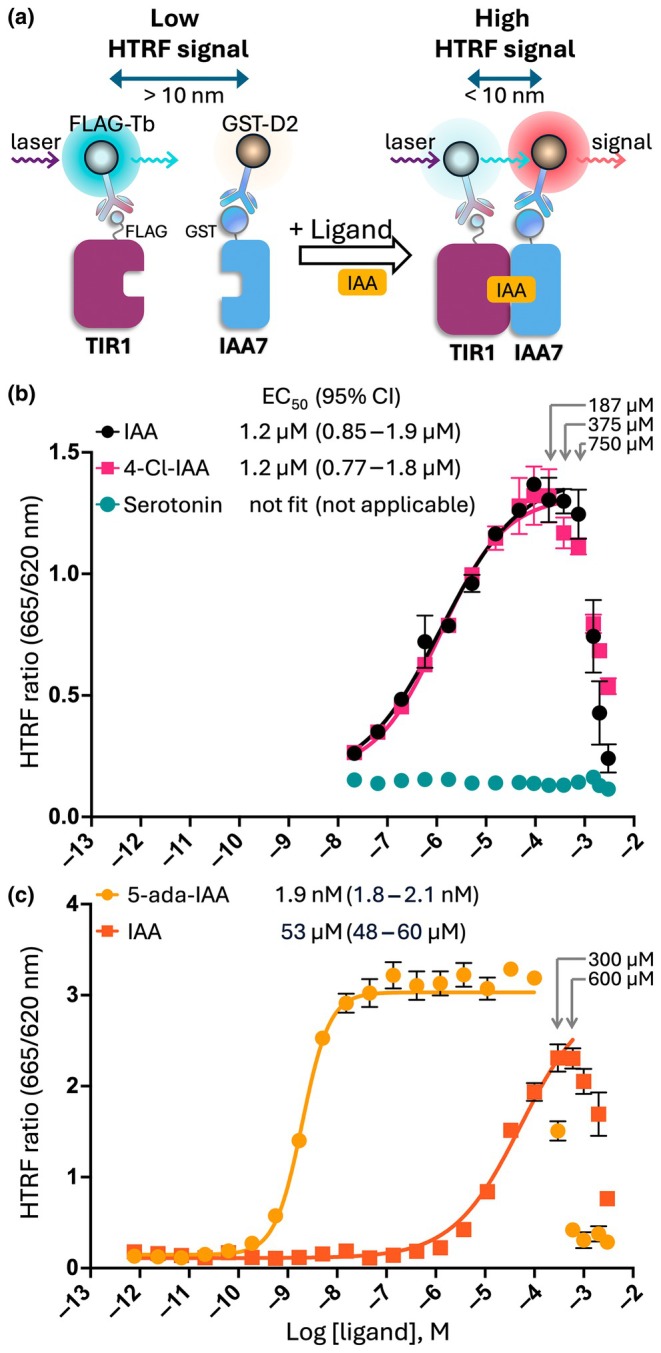
Homogeneous time‐resolved fluorescence (HTRF) assay to observe the binding between TRANSPORT INHIBITOR RESPONSE 1 (TIR1) and indole‐3‐acetic acid 7 (IAA7). (a) Schematic illustration of the HTRF assay. FLAG‐TIR1 is coupled with anti‐FLAG‐Tb (the donor), while the GST‐IAA7 is coupled with anti‐GST‐D2 (the acceptor). The presence of auxin induces the binding between TIR1 and IAA7, bringing the two fluorophores into proximity (< 10 nm) and releasing a high HTRF signal. (b, c) Concentration–response curves are used to determine the working concentration range of the ligand. Protein concentration was set at 25 nM each and incubated at 20°C for an hour before measurement. (b) Auxin (IAA) and 4‐chloro‐auxin (4‐Cl‐IAA) were used as positive controls, and serotonin as a negative control. The calculated EC_50_ and profile likelihood 95% confidence interval (95% CI) for the data points up to 375 μM are shown. (c) HTRF assay was performed using the TIR1^F79A^ variant and IAA7. The nonlinear regression for IAA was calculated by constraining the top value to be equal to the maximum value of 5‐Ada‐IAA. The calculated EC_50_ and 95% CI for the data points up to 100 μM for 5‐ada‐IAA and 600 μM for IAA are shown. Data are expressed as the mean ± SD of triplicates.

The HTRF technology has been widely used in many research fields. We therefore established an HTRF assay to quantitatively measure the binding between TIR1 and IAA7 coreceptors in the presence of auxin ligand (Fig. 2a). We first determined the effective working concentration range of the auxin ligands. We used IAA and 4‐Cl‐IAA, two forms of the naturally active auxins, and serotonin, an indoleamine compound possessing a similar chemical structure to auxin. Our study agreed with the previous study showing the lack of auxin activity of serotonin (Pelagio‐Flores *et al*., 2011). By contrast, a positive correlation was observed between the HTRF signal and the concentration of IAA or 4‐Cl‐IAA, with the signal reaching a maximum at *c*. 100 μM (Fig. 2b). Although 4‐Cl‐IAA has been reported to show more vigorous activity *in planta* (Karcz, 2002; Jayasinghege *et al*., 2019), IAA and 4‐Cl‐IAA showed identical potency to induce interaction between TIR1 and IAA7 in our study. For the data points up to 375 μM, the EC_50_ values were calculated to be 1.2 μM for both IAA and for 4‐Cl‐IAA.

The HTRF signals were sharply reduced as the concentration exceeded 750 μM and returned to the baseline at a higher concentration (3000 μM), producing a bell‐shaped curve. Considering the solubility of auxins, the reduced HTRF signal at high concentrations is likely due to ligand precipitation. To assess the limitation of the HTRF assay in the presence of excessive ligand, we used the 5‐ada‐IAA, which induces binding between TIR1^F79A^ and IAA proteins at a much lower concentration (Yamada *et al*., 2018). The maximum HTRF signal of 5‐ada‐IAA was detected at 10 nM and remained on a plateau up to 100 μM (Fig. 2c). The HTRF signal then sharply dropped at 300 μM and almost reached the baseline at higher concentrations. We noticed a visible precipitation of 5‐ada‐IAA above 600 μM (Fig. S2), which correlated with the reduced HTRF signal (Fig. 2c). The calculated EC_50_ of 5‐ada‐IAA for the data points up to 100 μM was 1.9 nM, comparable to the result from the ELISA. Overall, HTRF could serve as an alternative technique to quantify the auxin‐induced interactions between TIR1 and its coreceptor, sharing the common limitation of ligand solubility with other established methods.

### Optimizing the HTRF assay to evaluate the binding between TIR1 and IAA7

**Fig. 3:**
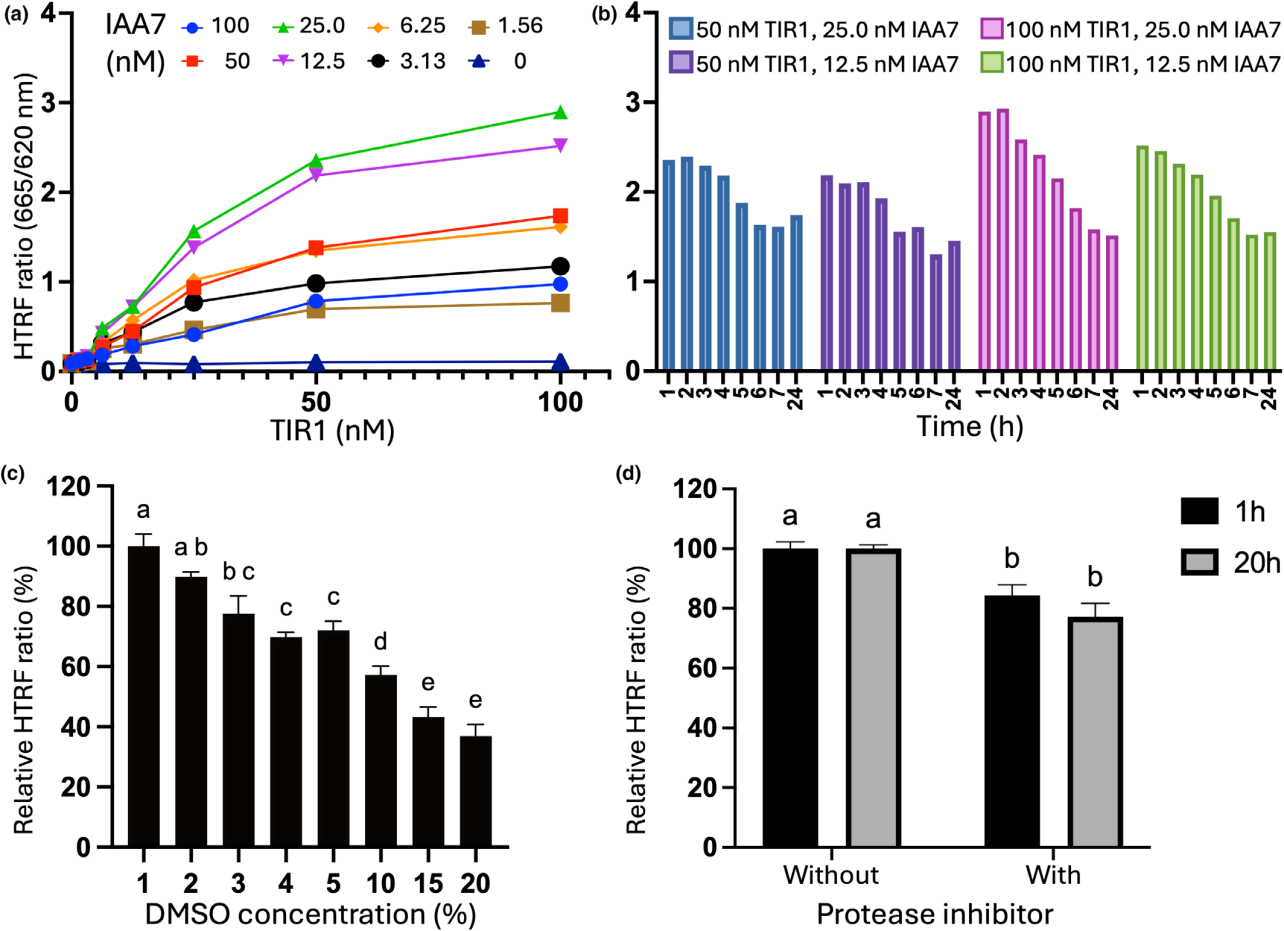
Optimizing the homogeneous time‐resolved fluorescence (HTRF) assay to evaluate the binding between TRANSPORT INHIBITOR RESPONSE 1 (TIR1) and indole‐3‐acetic acid 7 (IAA7). (a, b) Titration experiments to determine the optimal protein concentration of TIR1 and IAA7. (a) HTRF signal was measured after 1 h. (b) Time‐course analysis of the HTRF signal. Reaction was incubated at 22°C for up to 7 h, then stored at 4°C for a final measurement at 24 h. (c) The effect of DMSO concentration on the HTRF signal. (d) The effect of the protease inhibitor cocktail on the HTRF signal. Reactions were stored at 4°C for a final measurement at 20 h. Data are expressed as the mean ± SD from a single experiment with triplicate points. Different letters indicate statistically significant differences (*P* < 0.01) as determined by one‐way (c) and two‐way (d) ANOVA with Tukey's multiple comparisons test with a single pooled variance.

**Fig. 4:**
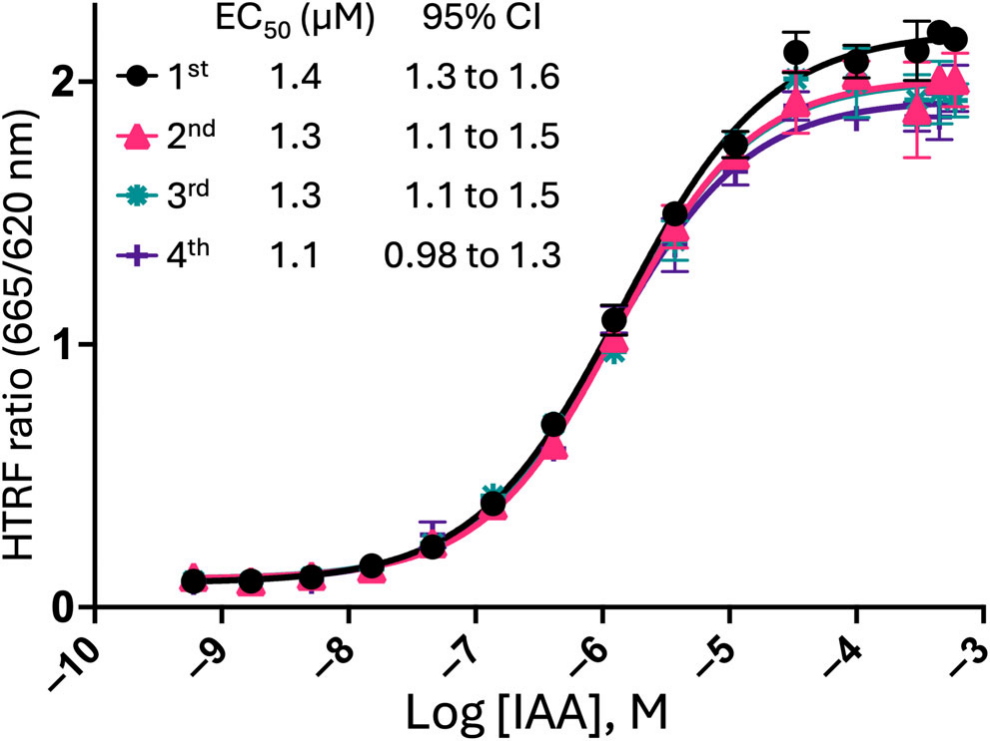
Evaluating the binding between TRANSPORT INHIBITOR RESPONSE 1 and indole‐3‐acetic acid 7 in the presence of auxin (IAA) using the homogeneous time‐resolved fluorescence assay. Data are expressed as the mean ± SD of triplicates from four experiments (*n* = 4). The geometric mean of the EC_50_ values across the four experiments was 1.3 μM.

We optimized the protein concentration for the HTRF assay using 150 μM IAA (Fig. 3a). A signal‐to‐background ratio of 25–33 was observed within the effective protein concentration range of 50–100 nM TIR1 and 12.5–25 nM IAA7. To efficiently use the limited supply of TIR1 protein, we fixed the protein concentration at 50 nM TIR1 and 25 nM IAA7 for our subsequent experiments. Signals remained stable for up to 3 h at room temperature (Fig. 3b). The HTRF signal was directly affected by the DMSO concentration (Fig. 3c). The addition of a protease inhibitor cocktail to the assay buffer reduced the HTRF signal by *c*. 20% (Fig. 3d). Even without the protease inhibitor, the HTRF signal was stable after overnight incubation at 4°C. Based on these results, we used the optimized conditions without the protease inhibitor and mixed the reaction on ice to evaluate the potency of auxin to induce the interaction between TIR1 and IAA7 (Fig. 4). The calculated EC_50_ ranged from 1.1 to 1.4 μM across four independent experiments, which is comparable to the results from experiments performed under suboptimal conditions (Fig. 2b). Consistent with these findings, WT TIR1 yielded comparable results, with a calculated EC_50_ value of 3.6 μM (Fig. S3).

### Evaluating the binding potency of auxin analogs using the HTRF assay

**Fig. 5:**
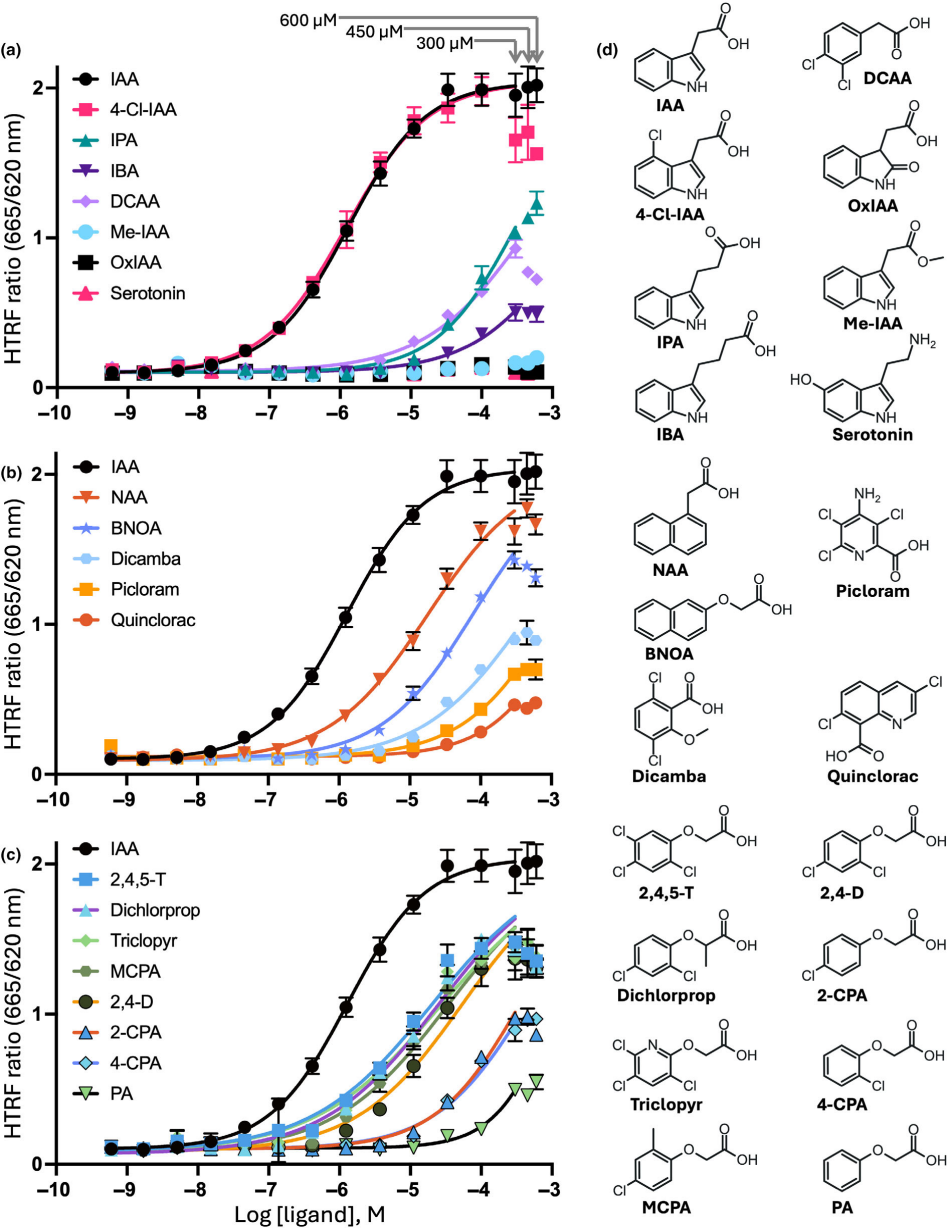
Homogeneous time‐resolved fluorescence assay to evaluate the potency of auxin analogs to induce the binding between TRANSPORT INHIBITOR RESPONSE 1 and indole‐3‐acetic acid 7. For easier visualization, the data are categorized as (a) IAA‐related, (b) 1‐naphthaleneacetic acid‐related (NAA) and benzoic acid‐related, and (c) phenoxyacetic acid‐related analogs. The same data points of auxin (IAA) are in all three panels. Non‐linear regression was calculated for data points up to 300 μM, with the top value constrained to the maximum response of IAA. Data are expressed as the mean standard deviation of triplicates. The calculated EC_50_ values are shown in Table 1. (d) Chemical structures of the auxin analogs evaluated in this study (details are listed in Supporting Information Table S1). Data are expressed as the mean ± SD of triplicates.

Using the optimized HTRF assay, we evaluated the binding potency of various auxin analogs (Fig. 5). Except for the 4‐Cl‐IAA, the tested auxin analogs did not reach the maximum binding value of IAA due to their low solubility in the aqueous solution. Therefore, the predicted EC_50_ was calculated by assuming that their maximum response would reach that of IAA.

We tested several naturally occurring plant‐derived indole derivatives, including indole‐3‐butyric acid (IBA), methyl indole‐3‐acetate (Me‐IAA), oxindole‐3‐acetic acid (OxIAA), and the microbe‐derived 3‐indolepropionic acid (IPA). IBA is considered a storage form of IAA (Korasick *et al*., 2013) that shows undetectable transcriptional and binding activity as an active auxin analog (Schlicht *et al*., 2013; Uzunova *et al*., 2016). Similar to the previous report using the SPR assay (Lee *et al*., 2014), we observed that IBA slightly induced the binding between TIR1 and IAA7 with the predicted EC_50_ value of 2.3 mM. Me‐IAA and OxIAA, which are inactive forms of auxin (Li *et al*., 2008; Yang *et al*., 2008; Peer *et al*., 2013), did not induce the binding between TIR1 and IAA7. Consistent with other studies using the SPR assay (Uzunova *et al*., 2016; Sun *et al*., 2024), IPA showed a relatively low potency with a predicted EC_50_ value of 300 μM.

We also evaluated some plant growth regulators with auxin‐like activities (Fig. 5; Table 1). 2‐Naphthoxyacetic acid (BNOA) has been reported as a virtually inactive auxin analog in the elongation zones of DR5rev::GFP roots at 1 μM (Scheitz *et al*., 2013). We similarly observed a minimal signal for BNOA at 1 μM, and its predicted EC_50_ value was determined to be 79 μM. Picloram and dicamba showed a relatively low potency, which is consistent with previous reports (Calderón Villalobos *et al*., 2012; Prusinska *et al*., 2022). We observed a very low potency of quinclorac, with a predicted EC_50_ value at 2.1 mM (Fig. 5b), which aligns with the previous report that quinclorac shows a very low root growth inhibition in *Arabidopsis* (Prusinska *et al*., 2022). 3,4‐Dichlorophenylacetic acid, known to have auxin activity in various crops (Tan *et al*., 2024), showed a predicted EC_50_ value of 480 μM. Phenoxyacetic acid (PA) derivatives are a class of synthetic auxins that are widely used in agriculture and research. Of all PA derivatives tested, 2,4,5‐trichlorophenoxyacetic acid (2,4,5‐T) showed the highest potency, with a predicted EC_50_ value of 20 μM. It was followed by: 2‐(2,4‐dichlorophenoxy)propionic acid (dichlorprop) at 24 μM; [(3,5,6‐trichloro‐2‐pyridinyl)oxy]acetic acid (triclopyr) at 27 μM; (4‐chloro‐2‐methylphenoxy)acetic acid (MCPA) at 34 μM; 2,4‐dichlorophenoxyacetic acid (2,4‐D) at 53 μM; 2‐chlorophenoxyacetic acid (2‐CPA) at 360 μM; 4‐chlorophenoxyacetic acid (4‐CPA) at 450 μM; and the PA itself at 1200 μM (Fig. 5c).

**Table 1:** Predicted half‐maximal effective concentration (EC_50_) values and 95% confidence interval (95% CI) of auxin analogs.

#	Ligand	EC_50_ (μM)	95% CI (μM)
1	IAA	1.3	1.2–1.4
2	4‐Cl‐IAA	1.2	0.9–1.5
3	IPA	300	270–340
4	IBA	2300	1600–3300
5	DCAA	480	420–570
6	Me‐IAA	Not fit	Not applicable
7	OxIAA	Not fit	Not applicable
8	Serotonin	Not fit	Not applicable
9	NAA	18	16–20
10	BNOA	79	73–86
11	Dicamba	470	400–550
12	Picloram	1100	890–1500
13	Quinclorac	2100	1600–2900
14	2,4,5‐T	20	17–24
15	Dichlorprop	24	20–29
16	Triclopyr	27	21–36
17	MCPA	34	28–40
18	2,4‐D	53	47–61
19	2‐CPA	360	320–400
20	4‐CPA	450	380–540
21	PA	1200	920–1500

### A mild increase in temperature impaired the binding of TIR1 to IAA7

**Fig. 6:**
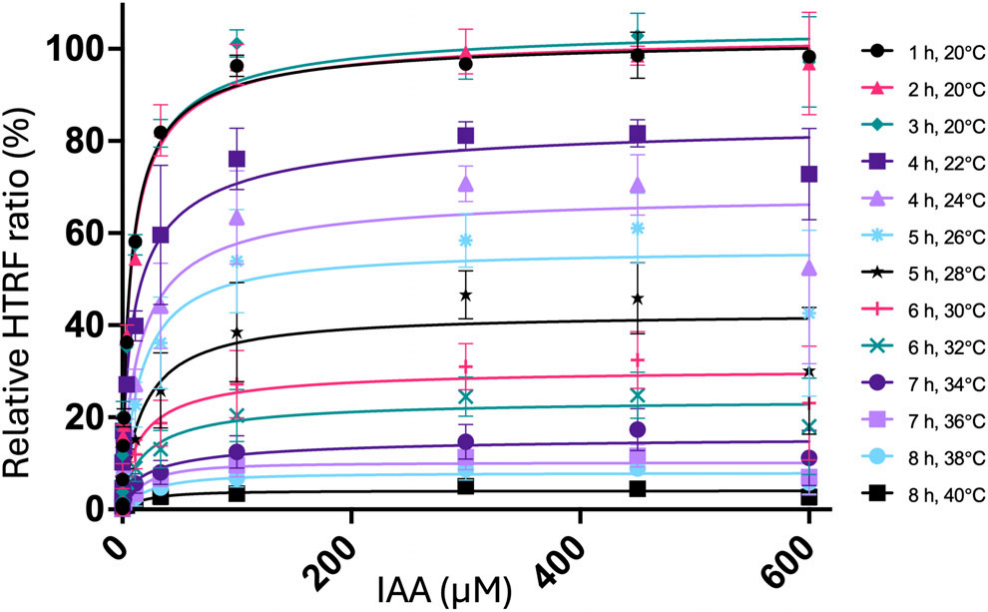
Homogeneous time‐resolved fluorescence (HTRF) assay to observe the binding between TRANSPORT INHIBITOR RESPONSE 1 and indole‐3‐acetic acid 7 at increasing temperatures. The reaction was initially incubated at 20°C and measured hourly for 3 h. It was then incubated at the indicated temperature for 10 min before being returned to 20°C for remeasurement. Data are expressed as the mean ± SD of a representative experiment (*n* = 2) performed in triplicate.

We observed a reduced HTRF signal over time at room temperature (*c*. 23°C), while the signal was stable after storing at 4°C (Fig. 3b,d). To investigate this further, we evaluated the effect of temperature on the binding between TIR1 and IAA7 using the HTRF assay. We incubated the reaction mixture containing TIR1, IAA7, protease inhibitors, and various concentrations of IAA at temperatures from 20°C to 40°C. A stable HTRF signal was observed during a 3‐h incubation at 20°C. By contrast, a 10‐min incubation at the higher temperatures gradually reduced the HTRF signal, which almost reached baseline after incubation at 40°C (Fig. 6). Consistent with these observations, comparable results were obtained using WT TIR1 (Fig. S4).

**Fig. 7:**
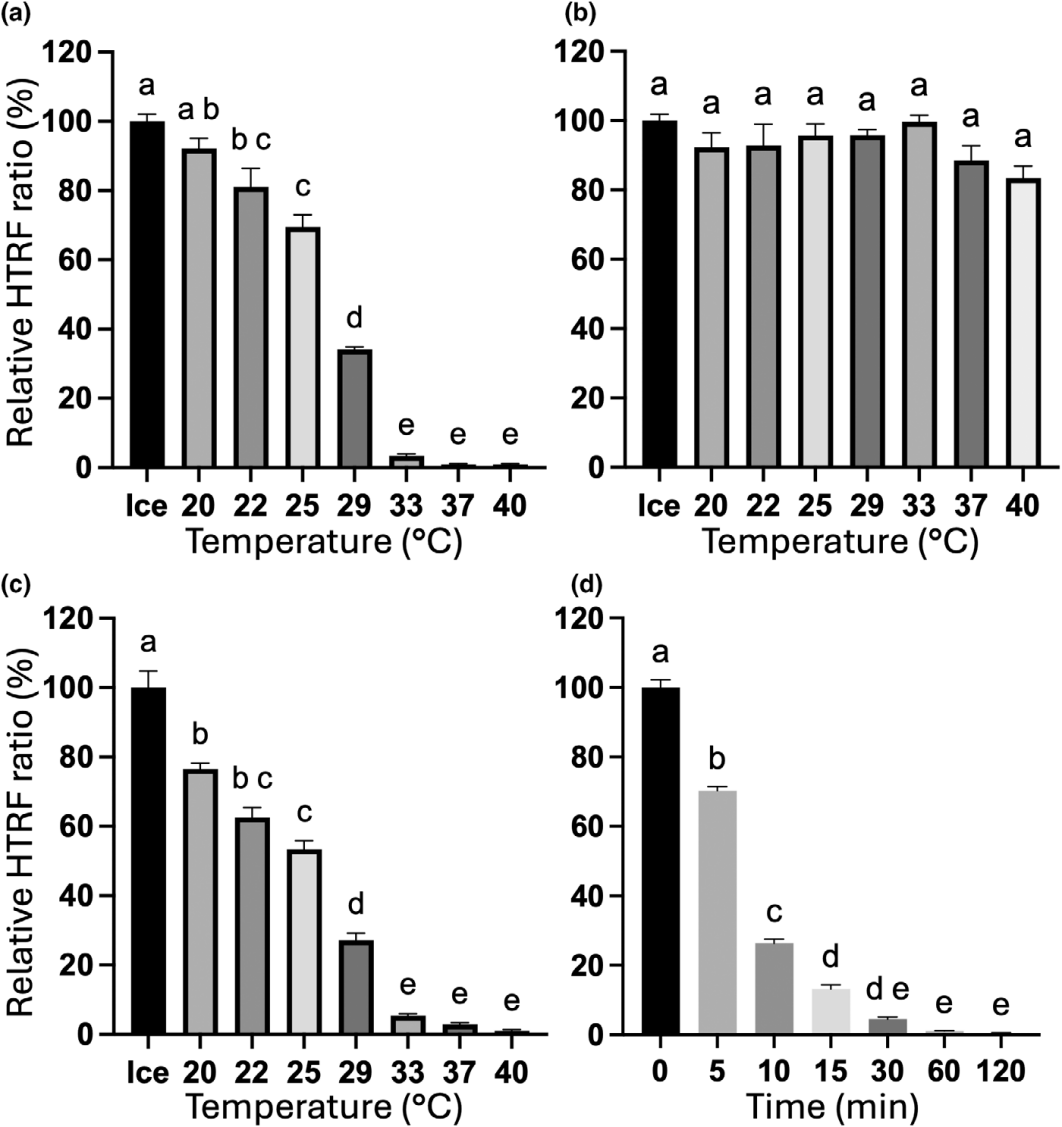
Homogeneous time‐resolved fluorescence assay of the pre‐incubated TRANSPORT INHIBITOR RESPONSE 1 (TIR1) or indole‐3‐acetic acid 7 (IAA7). (a) TIR1 pre‐incubation; (b) IAA7 pre‐incubation; (c) separate pre‐incubation of both TIR1 and IAA7 proteins. All proteins were pre‐incubated separately at the indicated temperature for 10 min, returned to ice for 1 h, and then mixed with other reaction components kept on ice. (d) TIR1 was pre‐incubated at 29°C for the indicated time, returned to ice until the end of incubation time (120 min), and then mixed with other reaction components kept on ice. Data are expressed as the mean with SE of the mean, from two independent experiments performed in triplicate. Different letters indicate statistically significant differences (*P* < 0.001) as determined by one‐way ANOVA with Tukey's multiple comparisons test with a single pooled variance.

To gain better insight, we separately pre‐incubated either TIR1, IAA7, or both proteins before mixing them with 150 μM IAA and other HTRF components that had been kept on ice (Fig. 7a–c). Pre‐incubating IAA7 did not significantly affect the HTRF signal (Fig. 7b), whereas pre‐incubating TIR1 significantly reduced the signal. Pre‐incubating TIR1 at 29°C for 10 min reduced the HTRF signal to less than half. Pre‐incubation at 33°C markedly reduced the signal to less than 6% (Fig. 7a,c). The HTRF signal was significantly reduced to 70% after 5 min, less than 5% after 30 min, and showed minimal binding at 1% after 1 h pre incubation at 29°C (Fig. 7d).

## Discussion

In this study, we developed an *in vitro* assay system to evaluate the binding between TIR1 and IAA7 auxin coreceptors. The half‐maximal effective concentration (EC_50_) of IAA to induce the binding between TIR1 and IAA7 was *c*. 1 μM in our study (0.81 μM in the ELISA and 1.3 μM in the HTRF assay). Our results are comparable to other studies evaluating the binding between TIR1 and IAA7‐degron‐peptide, in which reported EC_50_ values are 5 and 7.9 μM in the SPR assay and 2.7 μM in the AlphaScreen assays (Lee *et al*., 2014; Cao *et al*., 2022; Prusinska *et al*., 2022). As previously reported (Calderón Villalobos *et al*., 2012), the length of the IAA7 protein might explain the slight difference in the EC_50_ value between our study and others. We also found an EC_50_ of 5‐ada‐IAA at 1–2 nM for the interaction between TIR1^F79A^ and IAA7 in both ELISA and HTRF assays. Our results indicate that the HTRF‐binding assay is sufficiently sensitive to evaluate the interaction between TIR1 and IAA7. The mix‐and‐read format of the HTRF assay enables the rapid screening of a broad range of auxin analogs at a scale that is often impractical for SPR assays. Moreover, the time‐resolved aspect of HTRF reduces background interference from the tested compounds. While the AlphaScreen assay shares similar features with HTRF, the photobleaching of AlphaScreen donor beads effectively limits it to a single reading, making it less ideal for monitoring dynamic binding changes over time (Eglen *et al*., 2008). We have shown that the HTRF assay is effective for evaluating the potency of auxin analogs *in vitro*.

Elevated temperature has been linked to auxin signaling, which promotes hypocotyl elongation in *Arabidopsis* (Gray *et al*., 1998). About two decades ago, the instability of *A. thaliana* TIR1 was proposed during an *in vitro* pull‐down experiment at 37°C (Dharmasiri *et al*., 2005). Later, TIR1 was also reported to be less effective in the yeast cells, even at 24°C (Nishimura *et al*., 2009). Approximately a decade ago, it was reported that the chaperone HSP90 protein is required for TIR1 stabilization to respond at a mildly high temperature of 29°C *in planta* (Wang *et al*., 2016). Using the HTRF assay, which allowed multiple measurements over time, we observed that a mild increase in temperature impaired the binding between TIR1 and IAA7. Our subsequent experiments showed that temperature directly affected the binding activity of TIR1. Our results indicate that TIR1 is quite sensitive to a mild increase in temperature. The binding was not recovered by simply returning the protein to the ice, suggesting a structural deformation of the protein. Therefore, the rapid increase of TIR1 level upon incubation at 29°C (Wang *et al*., 2016) may occur to compensate for the loss of TIR1‐binding activity (Fig. 7d). Although future analyses are needed to understand how the increased temperature affects the binding activity of TIR1 at a structural level, our results agree with previous studies suggesting the instability of the *A. thaliana* TIR1 at increased temperatures. These data imply that TIR1 fulfills the criteria to be categorized as one of the potential heat‐sensing components in the plants (Vu *et al*., 2019), which is responsible for auxin signaling.

In conclusion, we have developed a robust *in vitro* system to evaluate the binding between TIR1 and IAA7 auxin coreceptors. We showed that our system was suitable for characterizing the potency of auxin analogs on a large scale and for gaining insight into the temperature sensitivity of the TIR1 protein. We expect our system to provide essential information for developing new auxin analogs with improved activity or for characterizing engineered auxin receptors. The system we presented in this study may also be applicable to evaluate other protein–protein interactions originating from plants, such as other plant hormone heterodimer coreceptors.

## Competing interests

None declared.

## Author contributions

JR performed the experiments. JR and SH designed the experiments. JR and SH analyzed the results. JR wrote the manuscript, and SH contributed to its review and editing.

## Disclaimer

The New Phytologist Foundation remains neutral with regard to jurisdictional claims in maps and in any institutional affiliations.

## Supporting information


**Fig. S1** SDS‐PAGE analysis of affinity‐purified proteins.
**Fig. S2** Precipitation of 5‐ada‐IAA and IAA in HTRF buffer.
**Fig. S3** Evaluating the binding between wild‐type TIR1 and IAA7 in the presence of auxin using the HTRF assay.
**Fig. S4** HTRF assay to observe the binding between wild‐type TIR1 and IAA7 at increasing temperatures.
**Notes S1** Maps of recombinant plasmids used in this study.
**Table S1** List of auxin analogs used in this study.
Please note: Wiley is not responsible for the content or functionality of any Supporting Information supplied by the authors. Any queries (other than missing material) should be directed to the *New Phytologist* Central Office.

## Data Availability

The data that support the findings of this study are available in the main text and Supporting Information Figs S1–S4, Table S1, and Notes S1 of this article.
