# Supplementary material for: Development of the HTRF assay to evaluate the auxin‐induced binding between TIR1 and IAA7
Source: New Phytol. 2026 Mar 11;250(5):3475–85. doi: 10.1111/nph.71084 (PMC13150301; doi:10.1111/nph.71084)
Supplement: Supplementary file 1 — Fig. S1 SDS‐PAGE analysis of affinity‐purified proteins. Fig. S2 Precipitation of 5‐ada‐IAA and IAA in HTRF buffer. Fig. S3 Evaluating the binding between wild‐type TIR1 and IAA7 in the presence of auxin using the HTRF assay. Fig. S4 HTRF assay to observe the binding between wild‐type TIR1 and IAA7 at increasing temperatures. Notes S1 Maps of recombinant plasmids used in this study. Table S1 List of auxin analogs used in this study. Please note: Wiley is not responsible for the content or functionality of any Supporting Information supplied by the authors. Any queries (other than missing material) should be directed to the New Phytologist Central Office. [file NPH-250-3475-s001.pdf]

New Phytologist

New Phytologist **Supporting Information**

Article title: **Development of the HTRF assay to evaluate the auxin-induced binding between TIR1 and IAA7**

Authors: Jekson Robertlee and Shinya Hagihara

RIKEN Center for Sustainable Resource Science, 2-1 Hirosawa, Wako, Saitama 351-0198, Japan

Article acceptance date: 18 February 2026

Figure S1. SDS-PAGE analysis of affinity-purified proteins.

Figure S2. Precipitation of 5-ada-IAA and IAA in HTRF buffer.

Figure S3. Evaluating the binding between wild-type TIR1 and IAA7 in the presence of auxin (IAA) using the HTRF assay.

Figure S4. HTRF assay to observe the binding between wild-type TIR1 and IAA7 at increasing temperatures.

Table S1. List of auxin analogs used in this study.

Supporting Notes. Maps of recombinant plasmids used in this study.

New Phytologist

The following Supporting Information is available for this article:

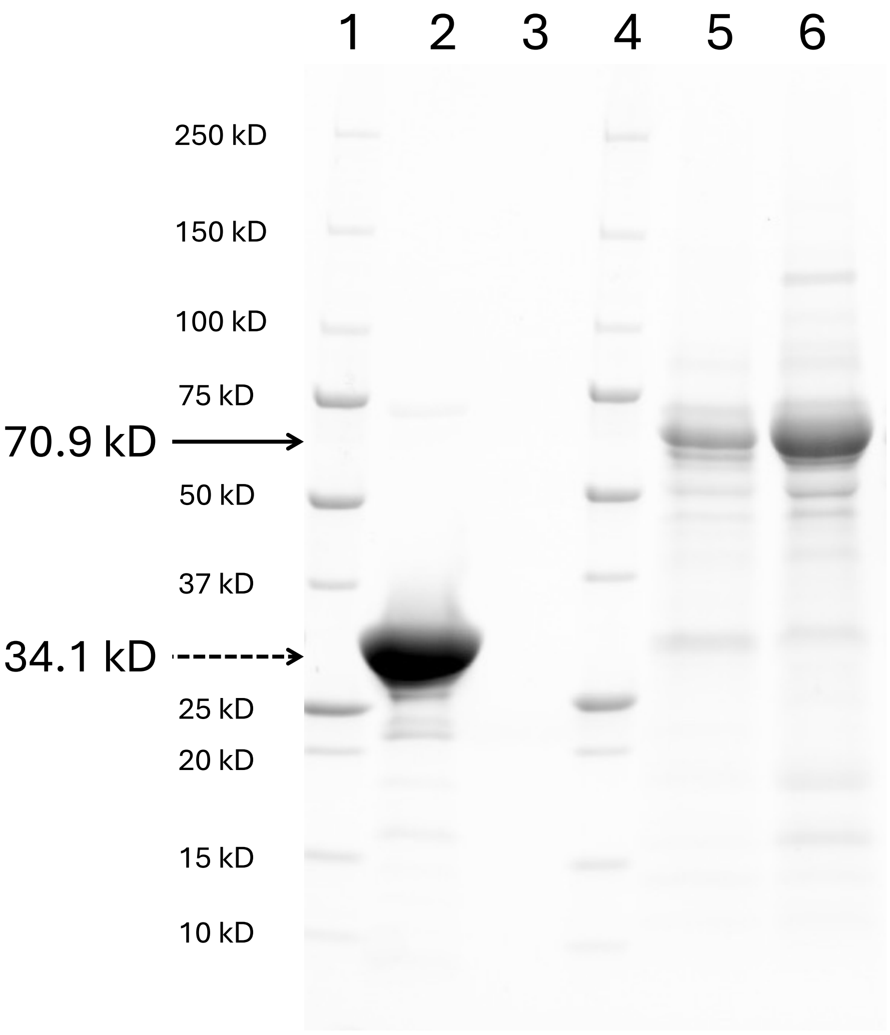

Fig. S1 SDS-PAGE analysis of the affinity-purified proteins.

Lane #1. Protein marker (BioRad #1610363)

Lane #2. GST_IAA7, protein with ~34.1 kD size

Lane #3. Empty lane

Lane #4. Protein marker (BioRad #1610363)

Lane #5. 6xHis_3xFLAG_AtTIR1-E12K-E15K-F79F, ~70.9 kD size

Lane #6. 6xHis_3xFLAG_AtTIR1-E12K-E15K-F79A, ~70.7 kD size

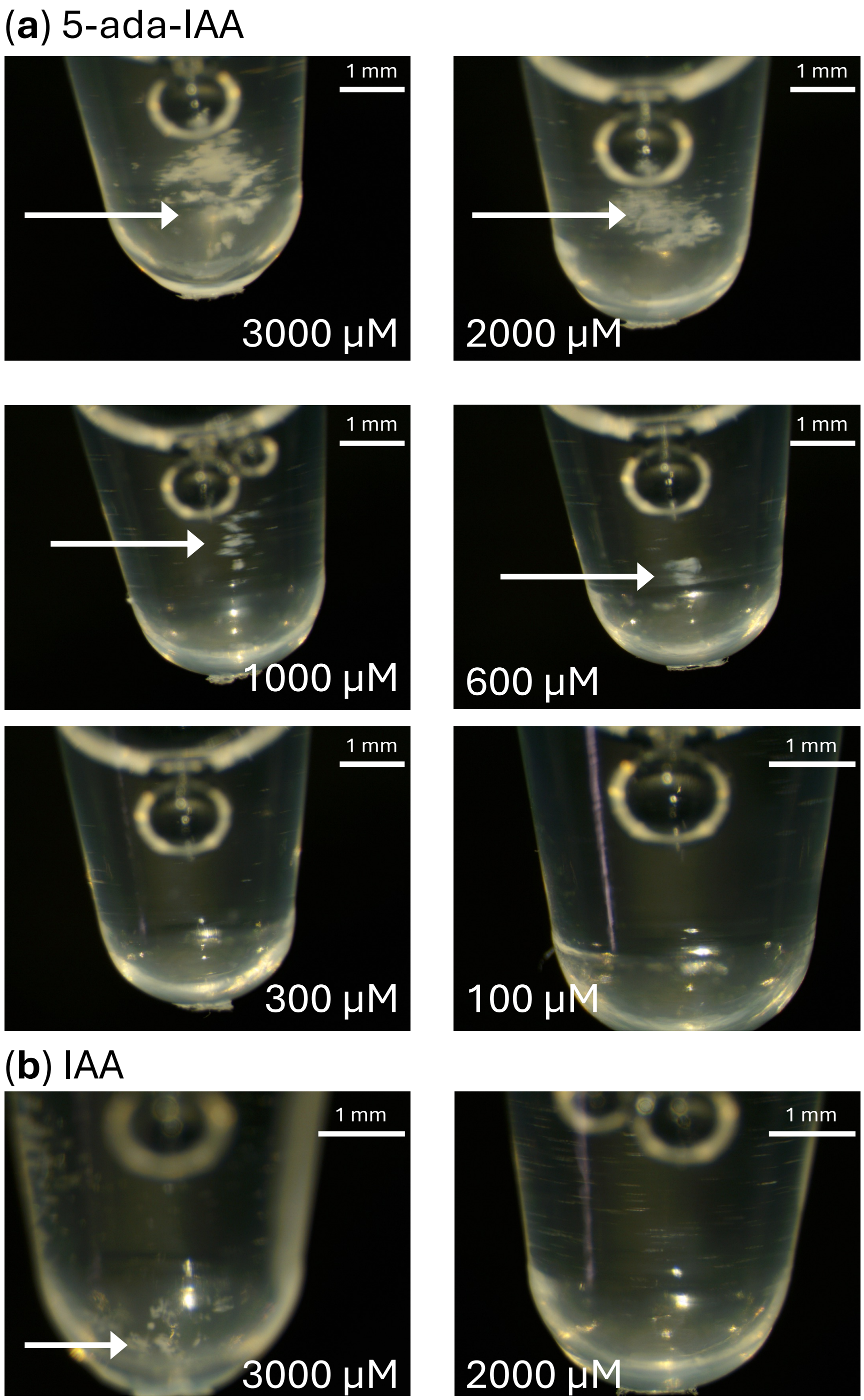

Fig. S2 Precipitation of 5-ada-IAA and IAA in HTRF buffer.

The compound (1% in DMSO) was added to the assay buffer (20 µL) without protein and HTRF reagents in the PCR tubes. The tubes were then observed using a stereo microscope at room temperature. (**a**) Precipitation was observed at 3000, 2000, 1000, and 600 µM; precipitation was not visible in 300 and 100 µM of 5-Ada-IAA. (**b**) Precipitation was observed at 3000 µM, while no visible precipitation was observed at 2000 µM of IAA.

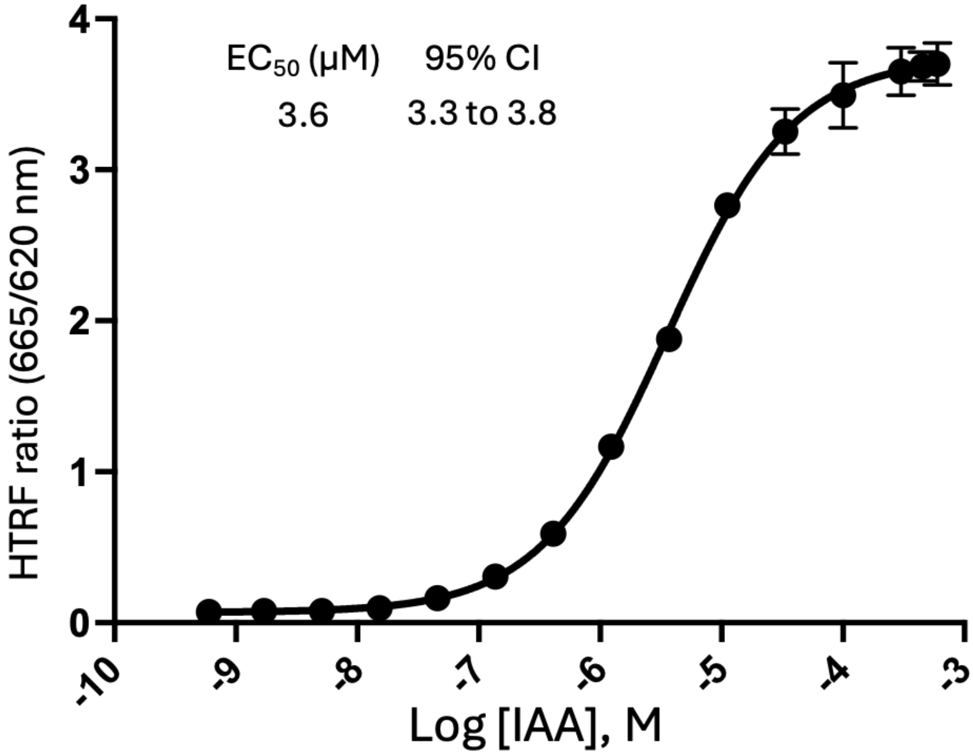

Fig. S3 Evaluating the binding between wild-type TIR1 and IAA7 in the presence of auxin (IAA) using the HTRF assay.

Data are expressed as the mean ± standard deviation of triplicates. The calculated EC_{50} and profile likelihood 95% confidence interval (95% CI) are shown.

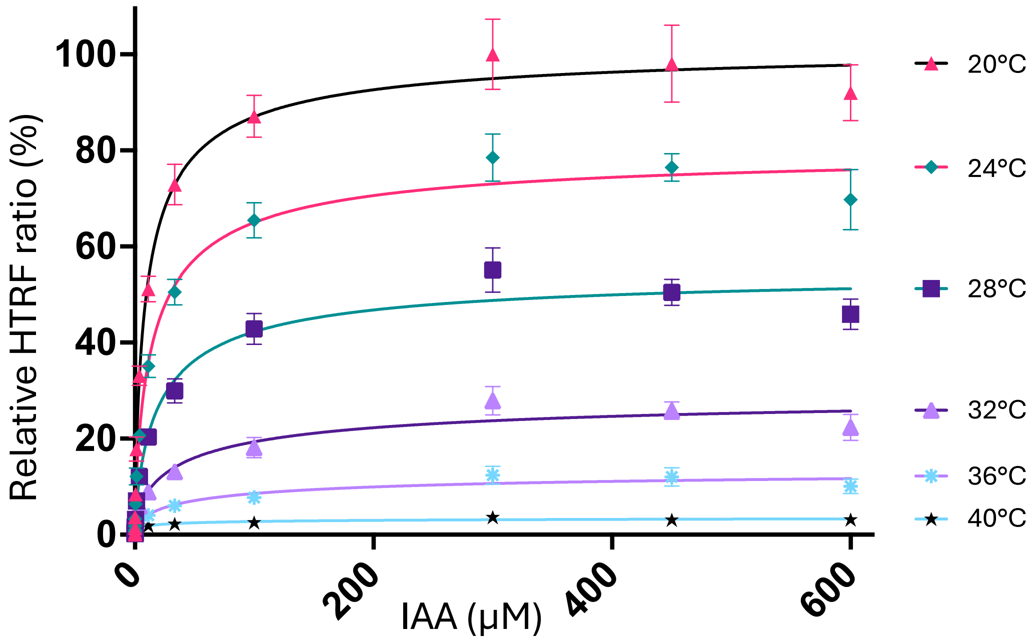

Fig. S4 HTRF assay to observe the binding between wild-type TIR1 and IAA7 at increasing temperatures.

The reaction was incubated at the indicated temperature for 10 minutes before being returned to 20°C for re-measurement. Data are expressed as the mean ± standard deviation performed in triplicate.

Table S1 List of auxin analogs used in this study.

#	Distributor	Ordering code	CAS RN:	Name	
1	TCI	I0022	87-51-4	IAA	3-Indoleacetic Acid
2	Sigma	AMBH2D7027F9	2519-61-1	4-Cl-IAA	4-Chloroindole-3-acetic acid
3	TCI	A3390	2244426-40-0	5-Ada-IAA	5-Adamantyl-IAA
4	TCI	I0032	830-96-6	IPA	3-Indolepropionic Acid
5	TCI	I0026	133-32-4	IBA	3-Indolebutyric Acid
6	TCI	M2605	1912-33-0	Me-IAA	Methyl Indole-3-acetate
7	Fujifilm	QM-4658	2971-31-5	OxIAA	Oxindole-3-acetic acid
8	Fujifilm	324-42331	50-67-9	Serotonin	Serotonin; 5-Hydroxytryptamine
9	TCI	N0005	86-87-3	NAA	1-Naphthaleneacetic Acid
10	TCI	N0045	120-23-0	BNOA	2-Naphthyloxyacetic Acid
11	Fujifilm	049-23573	1918-00-9	Dicamba	3,6-Dichloro-2-methoxybenzoic Acid
12	TCI	P1864	1918-02-1	Picloram	4-Amino-3,5,6-trichloropicolinic Acid
13	Fujifilm	F242893	84087-01-4	Quinclorac	3,7-Dichloroquinoline-8-carboxylic acid
14	Sigma	45667-250MG	93-76-5	2,4,5-T	2,4,5-Trichlorophenoxyacetic Acid
15	TCI	D1942	120-36-5	Dichlorprop	2-(2,4-Dichlorophenoxy)propionic Acid
16	TCI	T3742	55335-06-3	Triclopyr	[(3,5,6-Trichloro-2-pyridinyl)oxy]acetic Acid
17	TCI	C0206	94-74-6	MCPA	(4-Chloro-2-methylphenoxy)acetic Acid
18	TCI	D0396	94-75-7	2,4-D	2,4-Dichlorophenoxyacetic Acid
19	TCI	C0249	614-61-9	2-CPA	2-Chlorophenoxy acetic acid
20	TCI	C0250	122-88-3	4-CPA	4-Chlorophenoxyacetic Acid
21	TCI	D1813	5807-30-7	DCAA	3,4-Dichlorophenylacetic Acid
22	TCI	P0107	122-59-8	PA	Phenoxyacetic Acid

New Phytologist

Supporting Notes: Maps of recombinant plasmids used in this study.

pDGB3_alpha1 vector containing **pPAtUbq10, 6xHis_3xFLAG_TIR1-E12K-E15K-F79F, pTAtUbq3**

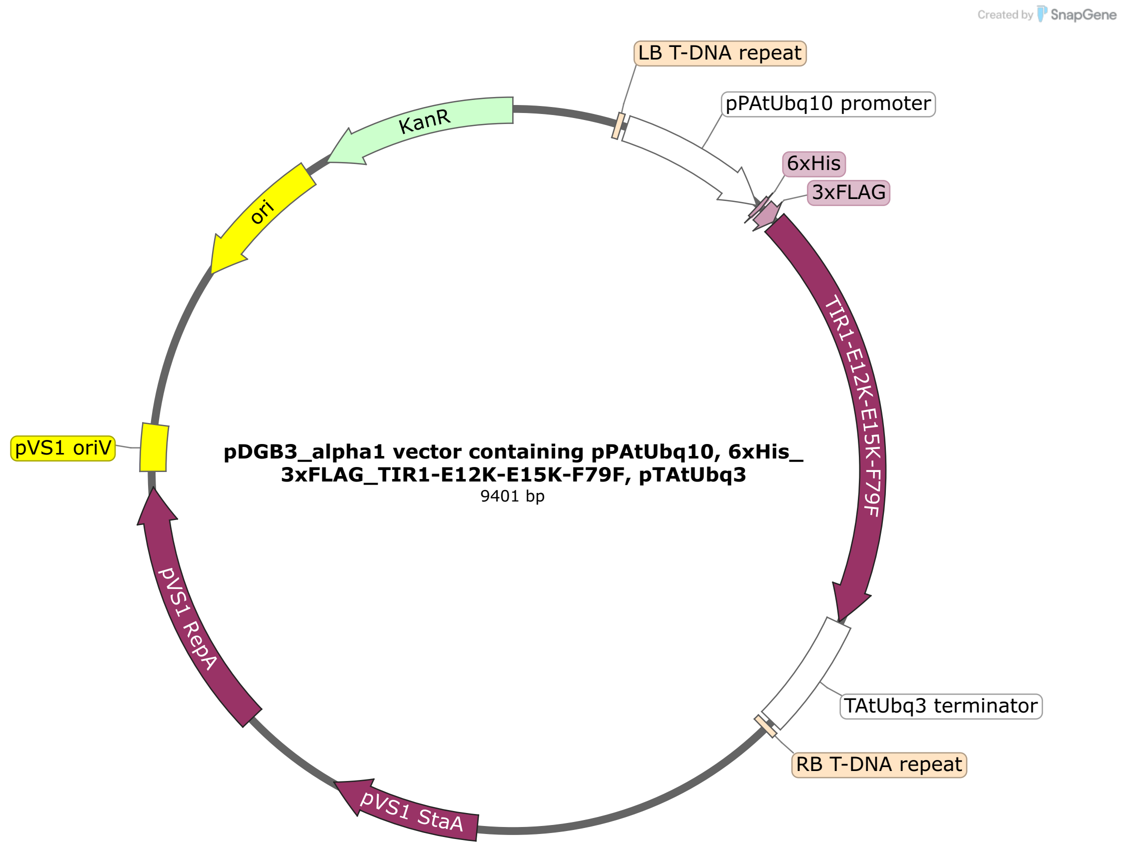

LB T-DNA repeat, pPAtUbq10 promoter, 6xHis_3xFLAG_TIR1-E12K-E15K-F79F, pTAtUbq3 terminator, RB T-DNA repeat

tggcaggatatattgtggtgtaaacataacgaattcgtctcaggaggtcgacgagtcagtaataaacggcgtcaaagtggttgcagccg
gcacacacgagtcgtgtttatcaactcaaagcacaaatacttttcctcaacctaaaaataaggcaattagccaaaaacaactttgcgtgt
aaacaacgctcaatacacgtgtcattttattattagctattgcttcaccgccttagctttctcgtgacctagtcgtcctcgtcttttcttctt
cttctataaaacaatacccaaagagctcttcttcttcacaattcagatttcaatttctcaaaatcttaaaaactttctctcaattctctctacc
gtgatcaaggtaaatttctgtgttccttattctctcaaaatcttcgattttgttttcgttcgatcccaatttcgtatatgttctttggtttagattc
tgttaatcttagatcgaagacgattttctgggtttgatcgttagatatcatcttaattctcgattagggtttcatagatatcatccgatttgttc
aaataatttgagttttgtcgaataattactcttcgatttgtgatttctatctagatctggtgttagtttctagtttgtgcgatcgaatttgtcgat
taatctgagtttttctgattaacagAATGCACCATCACCACCATCACGGTGATCAAGACTACAAGGATCATGATGG
GGACTATAAGGATCACGATATTGACTACAAAGATGACGATGACAAGGCAGCCATGCAGAAGCGAATAG
CCTTGTCGTTTCCAGAAAAGGTACTAAAGCATGTGTTCTCGTTTATTCAGCTGGATAAGGATAGGAACTC
AGTCTCTCTGGTGTGCAAGTCATGGTACGAGATCGAGCGGTGGTGCAGGAGGAAAGTCTTCATCGGGA

ACTGCTACGCCGTGAGTCCAGCGACGGTGATTAGGAGGTTCCCGAAAGTGAGATCCGTGGAGCTTAAA
GGAAAACCTCACTTTGCTGACTTTAATTTGGTACCTGACGGATGGGGAGGTTACGTGTATCCATGGATTG
AGGCCATGTCTTCGTCTTACACGTGGCTTGAAGAGATAAGGCTGAAGAGGATGGTGGTCACCGACGATT
GCTTGGAGCTCATAGCCAAGTCTTTTAAGAATTTTAAGGTACTAGTGCTTTCTTCCTGCGAAGGCTTCTCC
ACCGATGGTCTTGCTGCTATCGCTGCCACTTGCAGGAATCTGAAAGAGCTTGACTTACGAGAGAGTGAT
GTTGACGACGTTAGTGGCCACTGGCTTAGCCATTTCCCAGATACATACACTTCTTTGGTATCACTCAATAT
ATCTTGCTTAGCATCTGAGGTCAGTTTCTCTGCTCTGGAAAGGCTGGTGACTAGGTGTCCCAATCTCAAG
TCTCTCAAGCTTAACCGAGCTGTTCCACTTGAAAAATTGGCTACTTTACTTCAAAGAGCACCTCAATTGGA
GGAATTGGGCACTGGTGGGTACACTGCAGAAGTGCGACCAGATGTTTACTCTGGTTTATCTGTAGCGCT
CTCTGGGTGCAAGGAATTGAGGTGCTTATCTGGATTTTGGGATGCTGTTCCTGCCTATCTTCCAGCAGTT
TATTCGGTTTGCAGTCGGCTTACAACTTTGAATCTGAGTTATGCAACAGTCCAGAGCTATGATCTTGTCA
AGCTTCTTTGTCAATGCCCTAAACTGCAGCGCCTCTGGGTGCTTGACTACATCGAGGATGCTGGTCTTGA
GGTGCTTGCTTCAACCTGCAAGGACCTACGCGAGCTGAGAGTGTTTCCGTCCGAGCCTTTTGTCATGGAA
CCAAATGTGGCATTGACGGAACAGGGGCTTGTCTCCGTTTCCATGGGCTGTCCAAAACTCGAGTCGGTT
CTCTACTTCTGCCGTCAAATGACCAATGCTGCATTGATAACCATTGCTAGGAACCGTCCCAACATGACTC
GCTTCCGTTTGTGCATCATTGAGCCAAAAGCCCCAGACTATCTGACTCTAGAGCCACTGGATATTGGATT
TGGAGCCATAGTAGAGCACTGCAAGGATCTCCGTCGCCTCTCTCTATCTGGCCTCTTGACCGACAAGGTT
TTTGAATACATTGGGACATATGCCAAGAAGATGGAAATGCTCTCAGTGGCATTTGCAGGAGACAGTGAC
TTAGGCATGCATCATGTTTTGTCCGGGTGCGATAGCTTGAGGAAACTAGAGATAAGGGACTGCCCGTTT
GGAGACAAGGCGCTTTTGGCCAATGCTTCAAAGCTGGAGACAATGCGATCCCTTTGGATGTCTTCTTGTT
CCGTGAGTTTTGGAGCCTGCAAGTTACTAGGACAGAAGATGCCAAAGCTGAATGTGGAAGTCATCGAT
GAACGGGGTGCACCGGACTCGAGGCCAGAGAGCTGCCCTGTTGAGAGAGTCTTCATATACCGAACAGT
GGCTGGTCCTCGATTTGACATGCCTGGCTTCGTCTGGAACATGGACCAAGACTCAACAATGAGGTTTTCC
AGGCAAATCATTACTACTAACGGATTATAAgcttaagctttttgtgatctgatgataagtggttggttcgtgtctcatgcactt
gggaggtgatctatttcacctggtgtagtttgtgtttccgtcagttggaaaaacttatccctatcgatttcgttttcattttctgcttttcttttat
gtaccttcgtttgggcttgtaacgggcctttgtatttcaactctcaataataatccaagtgcatgttaaacaatttgtcatctgtttcggctttg
atatactactggtgaagatgggccgtactactgcatcacaacgaaaaataataataagatgaaaaacttgaagtggaaaaaaaaaaaa
cttgaatgttcactactactcattgaccataatgtttaacatacatagctcaatagtatttttgtgaatatggcaacacaaacagtccaaaa
caattgtctcttactataccaaaccaagggcgccgcttgtttgccactctttgtgtgcaatagtgtgattaccacacgctgtcatgagacga
attctgacaggatatattggcgggtaaac

pDGB3_alpha1 vector containing **pPAtUbq10, 6xHis_3xFLAG_TIR1-E12K-E15K-F79A, pTAtUbq3**

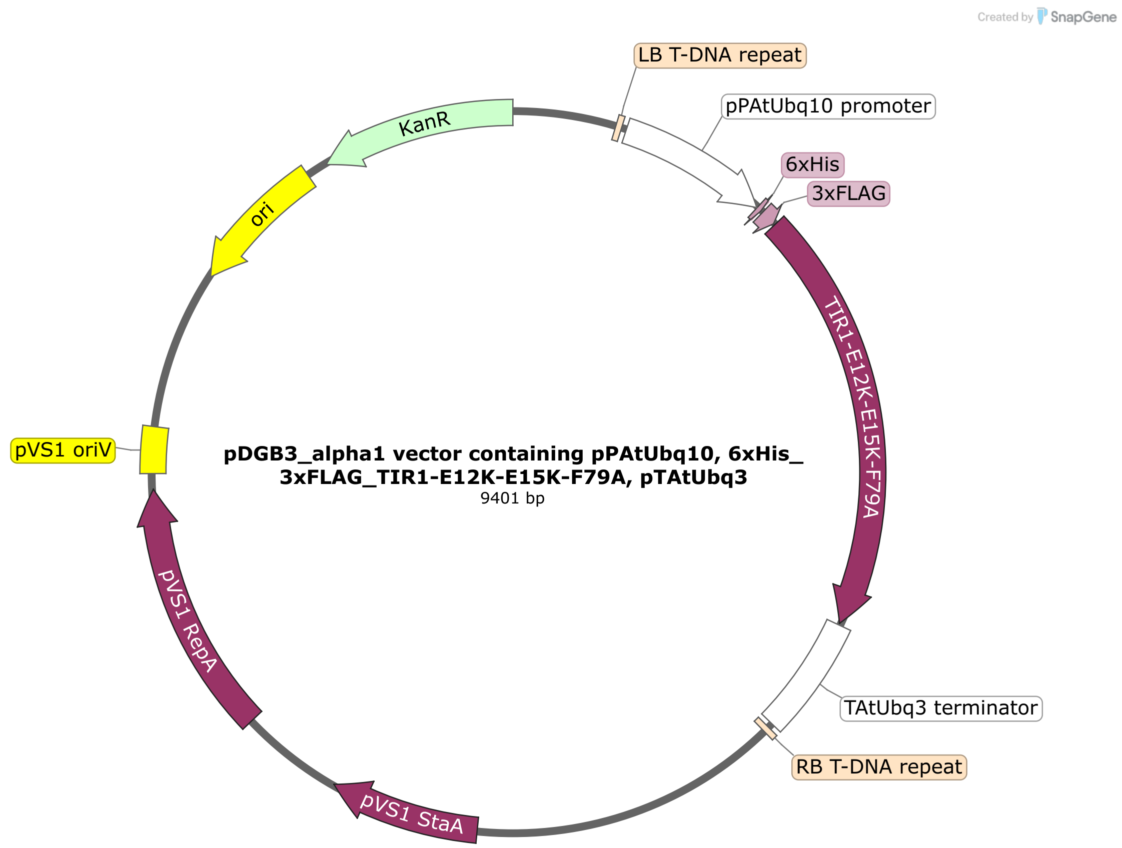

LB T-DNA repeat, pPAtUbq10 promoter, 6xHis_3xFLAG_TIR1-E12K-E15K-F79A, pTAtUbq3 terminator, RB T-DNA repeat

tggcaggatatattgtggtgtaaacataacgaattcgtctcaggaggtcgacgagtcagtaataaacggcgtcaaagtggttgcagccg
gcacacacgagtcgtgtttatcaactcaaagcacaaatacttttcctcaacctaaaaataaggcaattagccaaaaacaactttgcgtgt
aaacaacgctcaatacacgtgtcattttattattagctattgcttcaccgccttagctttctcgtgacctagtcgtcctcgtcttttcttcttcttc
cttctataaaacaatacccaaagagctcttcttcttcacaattcagatttcaatttctcaaaatcttaaaaactttctctcaattctctctacc
gtgatcaaggtaaatttctgtgttccttattctctcaaaatcttcgattttgttttcgttcgatcccaatttcgtatatgttctttggtttagattc
tgttaatcttagatcgaagacgattttctgggtttgatcgttagatatcatcttaattctcgattagggtttcatagatatcatccgatttgttc
aaataatttgagttttgtcgaataattactcttcgatttgtgatttctatctagatctggtgttagtttctagtttgtgcgatcgaatttgtcgat
taatctgagtttttctgattaacagAATGCACCATCACCACCATCACGGTGATCAAGACTACAAGGATCATGATGG
GGACTATAAGGATCACGATATTGACTACAAAGATGACGATGACAAGGCAGCCATGCAGAAGCGAATAG
CCTTGTCGTTTCCAGAAAAGGTACTAAAGCATGTGTTCTCGTTTATTCAGCTGGATAAGGATAGGAACTC
AGTCTCTCTGGTGTGCAAGTCATGGTACGAGATCGAGCGGTGGTGCAGGAGGAAAGTCTTCATCGGGA
ACTGCTACGCCGTGAGTCCAGCGACGGTGATTAGGAGGTTCCCGAAAGTGAGATCCGTGGAGCTTAAA
GGAAAACCTCACGCTGCTGACTTTAATTTGGTACCTGACGGATGGGGAGGTTACGTGTATCCATGGATT

GAGGCCATGTCTTCGTCTTACACGTGGCTTGAAGAGATAAGGCTGAAGAGGATGGTGGTCACCGACGA
TTGCTTGGAGCTCATAGCCAAGTCTTTTAAGAATTTTAAGGTACTAGTGCTTTCTTCCTGCGAAGGCTTCT
CCACCGATGGTCTTGCTGCTATCGCTGCCACTTGCAGGAATCTGAAAGAGCTTGACTTACGAGAGAGTG
ATGTTGACGACGTTAGTGGCCACTGGCTTAGCCATTTCCCAGATACATACACTTCTTTGGTATCACTCAAT
ATATCTTGCTTAGCATCTGAGGTCAGTTTCTCTGCTCTGGAAAGGCTGGTGACTAGGTGTCCCAATCTCA
AGTCTCTCAAGCTTAACCGAGCTGTTCCACTTGAAAAATTGGCTACTTTACTTCAAAGAGCACCTCAATTG
GAGGAATTGGGCACTGGTGGGTACACTGCAGAAGTGCGACCAGATGTTTACTCTGGTTTATCTGTAGCG
CTCTCTGGGTGCAAGGAATTGAGGTGCTTATCTGGATTTTGGGATGCTGTTCCTGCCTATCTTCCAGCAG
TTTATTCGGTTTGCAGTCGGCTTACAACTTTGAATCTGAGTTATGCAACAGTCCAGAGCTATGATCTTGTC
AAGCTTCTTTGTCAATGCCCTAAACTGCAGCGCCTCTGGGTGCTTGACTACATCGAGGATGCTGGTCTTG
AGGTGCTTGCTTCAACCTGCAAGGACCTACGCGAGCTGAGAGTGTTTCCGTCCGAGCCTTTTGTCATGGA
ACCAAATGTGGCATTGACGGAACAGGGGCTTGTCTCCGTTTCCATGGGCTGTCCAAAACTCGAGTCGGT
TCTCTACTTCTGCCGTCAAATGACCAATGCTGCATTGATAACCATTGCTAGGAACCGTCCCAACATGACTC
GCTTCCGTTTGTGCATCATTGAGCCAAAAGCCCCAGACTATCTGACTCTAGAGCCACTGGATATTGGATT
TGGAGCCATAGTAGAGCACTGCAAGGATCTCCGTCGCCTCTCTCTATCTGGCCTCTTGACCGACAAGGTT
TTTGAATACATTGGGACATATGCCAAGAAGATGGAAATGCTCTCAGTGGCATTTGCAGGAGACAGTGAC
TTAGGCATGCATCATGTTTTGTCCGGGTGCGATAGCTTGAGGAAACTAGAGATAgGGGACTGCCCGTTT
GGAGACAAGGCGCTTTTGGCCAATGCTTCAAAGCTGGAGACAATGCGATCCCTTTGGATGTCTTCTTGTT
CCGTGAGTTTTGGAGCCTGCAAGTTACTAGGACAGAAGATGCCAAAGCTGAATGTGGAAGTCATCGAT
GAACGGGGTGCACCGGACTCGAGGCCAGAGAGCTGCCCTGTTGAGAGAGTCTTCATATACCGAACAGT
GGCTGGTCCTCGATTTGACATGCCTGGCTTCGTCTGGAACATGGACCAAGACTCAACAATGAGGTTTTCC
AGGCAAATCATTACTACTAACGGATTATAAgcttaagctttttgtgatctgatgataagtggttggttcgtgtctcatgcactt
gggaggtgatctatttcacctggtgtagtttgtgtttccgtcagttggaaaaacttatccctatcgatttcgttttcattttctgcttttcttttat
gtaccttcgtttgggcttgtaacgggcctttgtatttcaactctcaataataatccaagtgcatgttaaacaatttgtcatctgtttcggctttg
atatactactggtgaagatgggccgtactactgcatcacaacgaaaaataataataagatgaaaaacttgaagtggaaaaaaaaaaaa
cttgaatgttcactactactcattgaccataatgtttaacatacatagctcaatagtatttttgtgaatatggcaacacaaacagtccaaaa
caattgtctcttactataccaaaccaagggcgccgcttgtttgccactctttgtgtgcaatagtgtgattaccacacgctgtcatgagacga
attctgacaggatatattggcgggtaaac

pDGB3_alpha1 vector containing **pPAtUbq10, pP19, pTAtUbq3**

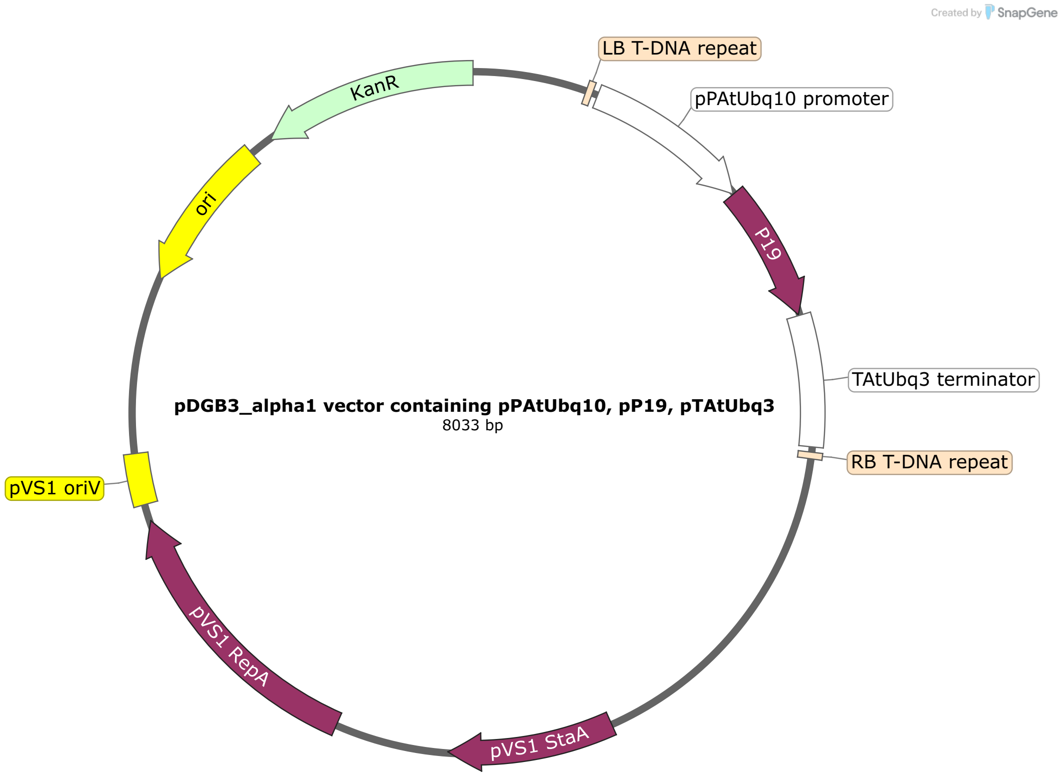

LB T-DNA repeat, pPAtUbq10 promoter, P19, pTAtUbq3 terminator, RB T-DNA repeat

tggcaggatatattgtggtgtaaacataacgaattcgtctcaggaggtcgacgagtcagtaataaacggcgtcaaagtggttgcagccg
gcacacacgagtcgtgtttatcaactcaaagcacaaatacttttcctcaacctaaaaataaggcaattagccaaaaacaactttgcgtgt
aaacaacgctcaatacacgtgtcattttattattagctattgcttcaccgccttagctttctcgtgacctagtcgtcctcgtcttttcttcttctt
cttctataaaacaatacccaaagagctcttcttcttcacaattcagatttcaatttctcaaaatcttaaaaactttctctcaattctctctacc
gtgatcaaggtaaatttctgtgttccttattctctcaaaatcttcgattttgttttcgttcgatcccaatttcgtatatgttctttggtttagattc
tgttaatcttagatcgaagacgattttctgggtttgatcgttagatatcatcttaattctcgattagggtttcatagatatcatccgatttgttc
aaataatttgagttttgtcgaataattactcttcgatttgtgatttctatctagatctggtgttagtttctagtttgtgcgatcgaatttgtcgat
taatctgagtttttctgattaacagaatggaacgagctatacaaggaaacgacgctagggaacaagctaacagtgaacgttgggatgga
ggatcaggaggtaccacttctcccttcaaacttcctgacgaaagtccgagttggactgagtggcggctacataacgatgagactaattcg
aatcaagataatccccttggtttcaaggaaagctggggtttcgggaaagttgtatttaagagatatctcagatacgacaggacggaagct
tcactgcacagagtccttggatcttggacgggagattcggttaactatgcagcatctcgatttttcggtttcgaccagatcggatgtacctat
agtattcggtttcgaggagttagtatcaccgtttctggaggctctcgaactcttcagcatctctgtgagatggcaattcggtctaagcaaga
actgctacagcttgccccaatcgaagtggaaagtaatgtatcaagaggatgccctgaaggtactgaaaccttcgaaaaagaaagcgagt
gagcttaagctttttgtgatctgatgataagtggttggttcgtgtctcatgcacttgggaggtgatctatttcacctggtgtagtttgtgtttcc
gtcagttggaaaaacttatccctatcgatttcgttttcattttctgcttttcttttatgtaccttcgtttgggcttgtaacgggcctttgtatttca
actctcaataataatccaagtgcatgttaaacaatttgtcatctgtttcggctttgatatactactggtgaagatgggccgtactactgcatc
acaacgaaaaataataataagatgaaaaacttgaagtggaaaaaaaaaaaacttgaatgttcactactactcattgaccataatgttta
acatacatagctcaatagtatttttgtgaatatggcaacacaaacagtccaaaacaattgtctcttactataccaaaccaagggcgccgct
tgtttgccactctttgtgtgcaatagtgtgattaccacacgctgtcatgagacgaattctgacaggatatattggcgggtaaac

pGEX-2T vector containing **GST_IAA7**

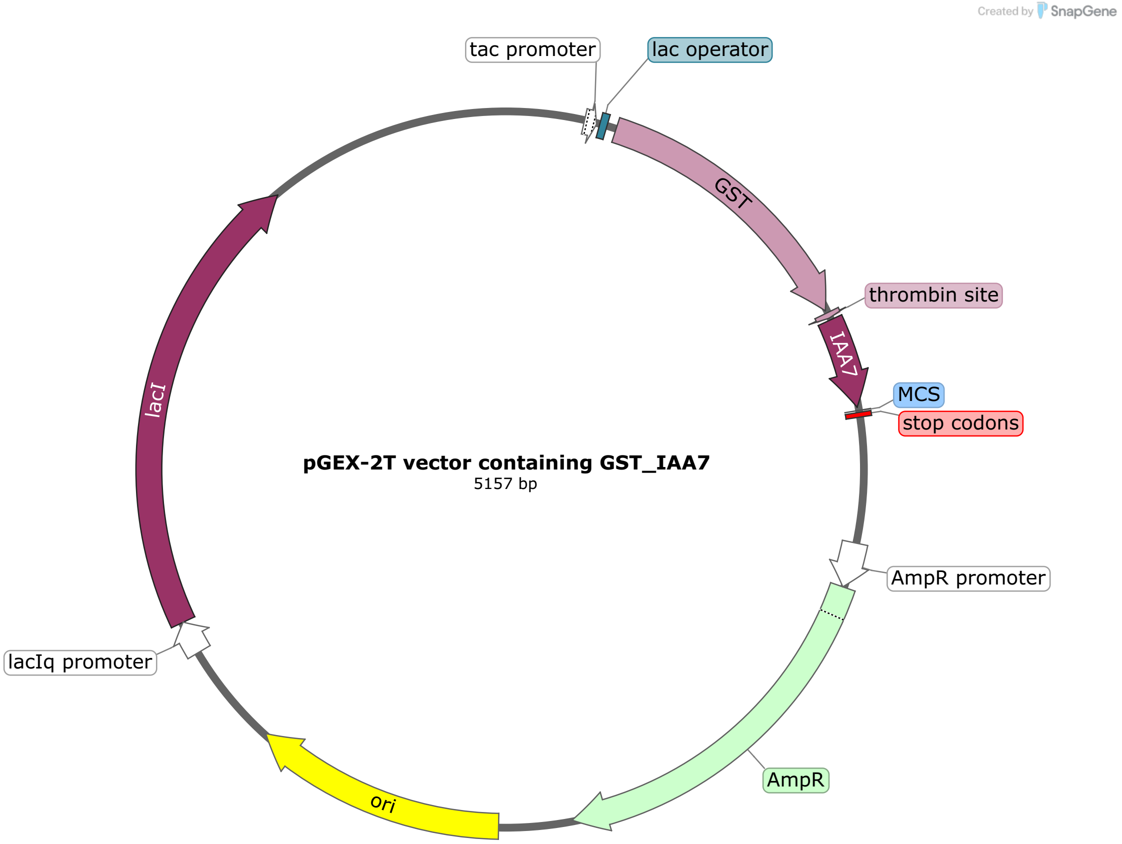

GST-tag_thrombin-site_IAA7

TTGACAATTAATCATCGGCTCGTATAATGTGTGGAATTGTGAGCGGATAACAATTTCACACAGGAAACA
GTATTCATGTCCCCTATACTAGGTTATTGGAAAATTAAGGGCCTTGTGCAACCCACTCGACTTCTTTTGGA
ATATCTTGAAGAAAAATATGAAGAGCATTTGTATGAGCGCGATGAAGGTGATAAATGGCGAAACAAAA
AGTTTGAATTGGGTTTGGAGTTTCCCAATCTTCCTTATTATATTGATGGTGATGTTAAATTAACACAGTCT
ATGGCCATCATACGTTATATAGCTGACAAGCACAACATGTTGGGTGGTTGTCCAAAAGAGCGTGCAGAG
ATTTCAATGCTTGAAGGAGCGGTTTTGGATATTAGATACGGTGTTTCGAGAATTGCATATAGTAAAGACT
TTGAAACTCTCAAAGTTGATTTTCTTAGCAAGCTACCTGAAATGCTGAAAATGTTCGAAGATCGTTTATGT
CATAAAACATATTTAAATGGTGATCATGTAACCCATCCTGACTTCATGTTGTATGACGCTCTTGATGTTGT
TTTATACATGGACCCAATGTGCCTGGATGCGTTCCCAAAATTAGTTTGTTTTAAAAAACGTATTGAAGCT
ATCCCACAAATTGATAAGTACTTGAAATCCAGCAAGTATATAGCATGGCCTTTGCAGGGCTGGCAAGCC
ACGTTTGGTGGTGGCGACCATCCTCCAAAATCGGATCTGGTTCCGCGTGgatccAAGAGAGGCTTCTCCG
AAACCGTTGATCTCATGCTCAATCTTCAATCTAACAAAGAAGGCTCCGTTGATCTCAAAAACGTTTCTGCT
GTTCCCAAGGAGAAGACTACCCTTAAAGATCCTTCTAAGCCTCCTGCTAAAGCACAAGTGGTGGGATGG
CCACCTGTGAGGAACTACAGGAAGAACATGATGACTCAGCAGAAGACCAGTAGTTGAgaattCATCGTGA
CTGACTGACGATCTGCCTCGCGCGTTTCGGTGATGACGG

pDGB3_alpha1 vector containing pPAtUbq10, 6xHis_3xFLAG_TIR1-E12E-E15E-F79F, pTAtUbq3

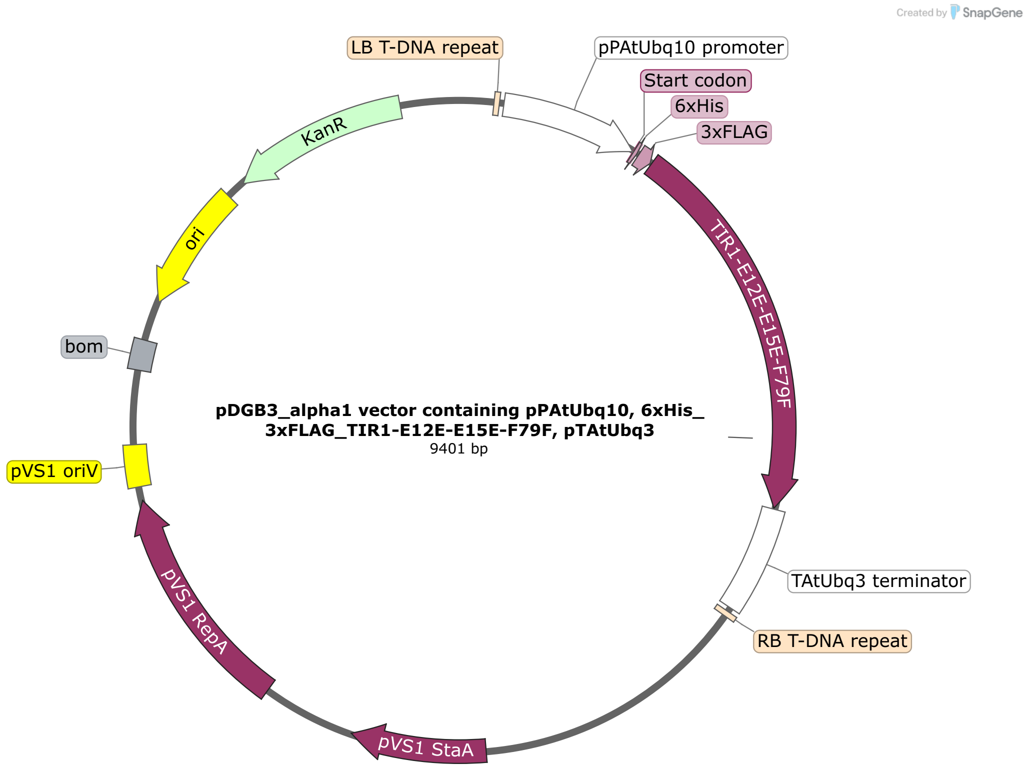

LB T-DNA repeat, pPAtUbq10 promoter, 6xHis_3xFLAG_TIR1-E12K-E15K-F79F, pTAtUbq3 terminator, RB T-DNA repeat

tggcaggatatattgtggtgtaaacataacgaattcgtctcaggaggtcgacgagtcagtaataaacggcgtcaaagtggttgcagccg
gcacacacgagtcgtgtttatcaactcaaagcacaaatacttttcctcaacctaaaaataaggcaattagccaaaaacaactttgcgtgt
aaacaacgctcaatacacgtgtcattttattattagctattgcttcaccgccttagctttctcgtgacctagtcgtcctcgtcttttcttctt
cttctataaaacaatacccaaagagctcttcttcttcacaattcagatttcaatttctcaaaatcttaaaaactttctctcaattctctctacc
gtgatcaaggtaaatttctgtgttccttattctctcaaaatcttcgattttgttttcgttcgatcccaatttcgtatatgttctttggtttagattc
tgttaatcttagatcgaagacgattttctgggtttgatcgttagatatcatcttaattctcgattagggtttcatagatatcatccgatttgttc
aaataatttgagttttgtcgaataattactcttcgatttgtgatttctatctagatctggtgttagtttctagtttgtgcgatcgaatttgtcgat
taatctgagtttttctgattaacagAATGCACCATCACCACCATCACGGTGATCAAGACTACAAGGATCATGATGG
GGACTATAAGGATCACGATATTGACTACAAAGATGACGATGACAAGGCAGCCATGCAGAAGCGAATAG
CCTTGTCGTTTCCAGAAGAGGTACTAGAGCATGTGTTCTCGTTTATTCAGCTGGATAAGGATAGGAACTC
AGTCTCTCTGGTGTGCAAGTCATGGTACGAGATCGAGCGGTGGTGCAGGAGGAAAGTCTTCATCGGGA
ACTGCTACGCCGTGAGTCCAGCGACGGTGATTAGGAGGTTCCCGAAAGTGAGATCCGTGGAGCTTAAA

GGAAAACCTCACTTTGCTGACTTTAATTTGGTACCTGACGGATGGGGAGGTTACGTGTATCCATGGATTG
AGGCCATGTCTTCGTCTTACACGTGGCTTGAAGAGATAAGGCTGAAGAGGATGGTGGTCACCGACGATT
GCTTGGAGCTCATAGCCAAGTCTTTTAAGAATTTTAAGGTTCTTGTGCTTTCTTCCTGCGAAGGCTTCTCC
ACCGATGGTCTTGCTGCTATCGCTGCCACTTGCAGGAATCTGAAAGAGCTTGACTTACGAGAGAGTGAT
GTTGACGACGTTAGTGGCCACTGGCTTAGCCATTTCCCAGATACATACACTTCTTTGGTATCACTCAATAT
ATCTTGCTTAGCATCTGAGGTCAGTTTCTCTGCTCTGGAAAGGCTGGTGACTAGGTGTCCCAATCTCAAG
TCTCTCAAGCTTAACCGAGCTGTTCCACTTGAAAAATTGGCTACTTTACTTCAAAGAGCACCTCAATTGGA
GGAATTGGGCACTGGTGGGTACACTGCAGAAGTGCGACCAGATGTTTACTCTGGTTTATCTGTAGCGCT
CTCTGGGTGCAAGGAATTGAGGTGCTTATCTGGATTTTGGGATGCTGTTCCTGCCTATCTTCCAGCAGTT
TATTCGGTTTGCAGTCGGCTTACAACTTTGAATCTGAGTTATGCAACAGTCCAGAGCTATGATCTTGTCA
AGCTTCTTTGTCAATGCCCTAAACTGCAGCGCCTCTGGGTGCTTGACTACATCGAGGATGCTGGTCTTGA
GGTGCTTGCTTCAACCTGCAAGGACCTACGCGAGCTGAGAGTGTTTCCGTCCGAGCCTTTTGTCATGGAA
CCAAATGTGGCATTGACGGAACAGGGGCTTGTCTCCGTTTCCATGGGCTGTCCAAAACTCGAGTCGGTT
CTCTACTTCTGCCGTCAAATGACCAATGCTGCATTGATAACCATTGCTAGGAACCGTCCCAACATGACTC
GCTTCCGTTTGTGCATCATTGAGCCAAAAGCCCCAGACTATCTGACTCTAGAGCCACTGGATATTGGATT
TGGAGCCATAGTAGAGCACTGCAAGGATCTCCGTCGCCTCTCTCTATCTGGCCTCTTGACCGACAAGGTT
TTTGAATACATTGGGACATATGCCAAGAAGATGGAAATGCTCTCAGTGGCATTTGCAGGAGACAGTGAC
TTAGGCATGCATCATGTTTTGTCCGGGTGCGATAGCTTGAGGAAACTAGAGATAAGGGACTGCCCGTTT
GGAGACAAGGCGCTTTTGGCCAATGCTTCAAAGCTGGAGACAATGCGATCCCTTTGGATGTCTTCTTGTT
CCGTGAGTTTTGGAGCCTGCAAGTTACTAGGACAGAAGATGCCAAAGCTGAATGTGGAAGTCATCGAT
GAACGGGGTGCACCGGACTCGAGGCCAGAGAGCTGCCCTGTTGAGAGAGTCTTCATATACCGAACAGT
GGCTGGTCCTCGATTTGACATGCCTGGCTTCGTCTGGAACATGGACCAAGACTCAACAATGAGGTTTTCC
AGGCAAATCATTACTACTAACGGATTATAAgcttaagctttttgtgatctgatgataagtggttggttcgtgtctcatgcactt
gggaggtgatctatttcacctggtgtagtttgtgtttccgtcagttggaaaaacttatccctatcgatttcgttttcattttctgcttttcttttat
gtaccttcgtttgggcttgtaacgggcctttgtatttcaactctcaataataatccaagtgcatgttaaacaatttgtcatctgtttcggctttg
atatactactggtgaagatgggccgtactactgcatcacaacgaaaaataataataagatgaaaaacttgaagtggaaaaaaaaaaa
cttgaatgttcactactactcattgaccataatgtttaacatacatagctcaatagtatttttgtgaatatggcaacacaaacagtccaaaa
caattgtctcttactataccaaaccaagggcgccgcttgtttgccactctttgtgtgcaatagtgtgattaccacacgctgtcatgagacga
attctgacaggatatattggcgggtaaac
